# METTL3 regulates PRRSV replication by suppressing interferon beta through autophagy-mediated IKKε degradation

**DOI:** 10.1128/jvi.00098-25

**Published:** 2025-06-23

**Authors:** Yunyun Zhai, Lucai Wang, Lijie Lv, Xuyang Zhao, Mengjie Li, Jiajing Tian, Xiangqi Qiu, Lulu Yao, Wenhui Zhu, Yunzhe Kang, Angke Zhang, Guoqing Zhuang, Aijun Sun

**Affiliations:** 1 International Joint Research Center of National Animal Immunology, College of Veterinary Medicine, Henan Agricultural University 731518, Zhengzhou, China; 2 Longhu Laboratory of Advanced Immunology 12636, Zhengzhou, China; 3 Ministry of Education Key Laboratory for Animal Pathogens and Biosafety, Henan Agricultural University 70573, Zhengzhou, China; University of Kentucky College of Medicine, Lexington, Kentucky, USA

**Keywords:** N6-methyladenosine (m^6^A), PRRSV, METTL3, IKKε, SQSTM1/p62, type I interferon

## Abstract

Methyltransferase-like-3 (METTL3)-mediated N6-methyladenosine (m^6^A) modification of messenger RNAs plays a pivotal role in regulating innate immune responses, either promoting or combating virus replication. However, the biological function of METTL3 during porcine reproductive and respiratory syndrome virus (PRRSV) infection remains unclear. In this study, we found that PRRSV infection reprograms m^6^A modifications in cellular transcripts, enhances METTL3 expression, and alters its subcellular distribution. Intriguingly, METTL3 overexpression facilitates PRRSV replication, whereas its deficiency suppresses it, primarily through the negative regulation of type I interferon (IFN-I) production. Further investigation revealed that METTL3 interacts with and promotes the degradation of IκB kinase-ε (IKKε) during PRRSV infection. Mechanistically, METTL3-mediated m^6^A modification of SQSTM1 (sequestosome 1) enhances *SQSTM1* messenger RNA (mRNA) expression, increasing autophagy levels. Moreover, METTL3 facilitates the formation of K63-linked ubiquitin chains on IKKε, targeting it for degradation via SQSTM1-dependent selective autophagy. Collectively, our findings unveil a novel mechanism whereby METTL3 facilitates PRRSV replication by suppressing antiviral innate immunity, thereby offering potential targets for antiviral therapy.

**IMPORTANCE:**

Porcine reproductive and respiratory syndrome (PRRS), induced by the porcine reproductive and respiratory syndrome virus (PRRSV), poses a highly contagious threat to the global swine industry, leading to substantial economic losses. The genetic variability and immune evasion capabilities of PRRSV complicate the development of effective vaccines and control strategies. Thus, a comprehensive understanding of PRRSV’s immune evasion mechanisms is imperative. In this study, we reveal that METTL3 plays a pivotal role in PRRSV’s evasion of interferon (IFN) immunity. Specifically, METTL3 targets IKKε, inducing its autophagy degradation and subsequently inhibiting the expression of interferon beta 1 (IFNB1). Furthermore, PRRSV infection alters the N6-methyladenosine (m^6^A) modification of various host genes, with notable changes observed in the m^6^A modification and transcriptional levels of SQSTM1, which are regulated by METTL3. This regulation is crucial for SQSTM1-mediated autophagy degradation of IKKε. Our findings offer novel insights into the mechanisms underlying host protein involvement in PRRSV’s immune evasion.

## INTRODUCTION

Porcine reproductive and respiratory syndrome (PRRS), caused by the porcine reproductive and respiratory syndrome virus (PRRSV), is characterized by reproductive failures in sows and respiratory distress in pigs of all ages, leading to substantial economic losses worldwide (1). PRRSV, classified under the genus *Betaarterivirus* in the family *Arteriviridae* and order *Nidovirales*, possesses a highly variable positive-strand RNA genome (2). Despite the use of inactivated and attenuated vaccines, PRRSV continues to evolve, evading host immune defenses (3). As an alternative, antiviral drugs targeting the interferon (IFN) pathway have shown promise in managing PRRSV infections (4). Recombinant IFNs, including interferon alpha, IFNB1, and interferon lambda, demonstrate potential for inhibiting PRRSV replication (5). Thus, unraveling the molecular mechanisms by which PRRSV manipulates the innate immune response is critical for developing effective vaccines and antiviral therapies.

The innate immune system, the first line of defense against pathogens, is activated through the recognition of pathogen-associated molecular patterns by pattern-recognition receptors (PRRs) (6). During RNA virus infections, RIG-I-like receptors—comprising RIG-I (retinoic acid-inducible gene I), MDA5 (melanoma differentiation-associated gene 5), and LGP2 (laboratory of genetics and physiology 2)—serve as key PRRs (7). Upon recognizing double-stranded RNA (dsRNA), RIG-I and MDA5 undergo conformational changes, interacting with the adaptor mitochondrial antiviral signaling (MAVS) protein (8). This interaction activates downstream kinases, including TANK-binding kinase 1 (TBK1) and IκB kinase-ε (IKKε), culminating in the production of IFN-I and inflammatory cytokines (9). PRRSV has developed diverse strategies to evade host immunity, including suppression of IFNB1 production via the autophagy pathway (10). Autophagy, an essential cellular process for degrading cellular or pathogenic components, includes selective autophagy, which specifically targets substrates through cargo receptors such as SQSTM1 (sequestosome 1), NBR1 (neighbor of BRCA1 gene 1), OPTN (optineurin), and TOLLIP (toll-interacting protein) (11). During PRRSV infection, the autophagy receptor SQSTM1 and chaperonin CCT2 mediate the degradation of MDA5, suppressing innate immune responses (12). PRRSV’s envelope protein E further inhibits DEAD-box helicase 10 (DDX10) antiviral activity by enhancing its interaction with SQSTM1, leading to DDX10 degradation (13). This intricate interplay underscores PRRSV’s ability to hijack host processes for its benefit. Despite extensive research, the molecular mechanisms underlying PRRSV infection and immune evasion remain incompletely understood.

N6-methyladenosine (m^6^A) is a common post-transcriptional modification of messenger RNA (mRNA) that regulates diverse biological and pathological processes (14). This modification is catalyzed by a methyltransferase complex comprising METTL3 (methyltransferase-like 3), METTL14 (methyltransferase-like 14), and WTAP (Wilms tumor 1-associated protein) (15–17), and is reversible by demethylases such as FTO (fat mass and obesity-associated protein) and ALKBH5 (alkB homolog 5) (18–20). m^6^A-modified RNAs are recognized by reader proteins, including YTHDF1/2/3, IGF2BP1/2/3, HNRNPA2B1, and HNRNPC, which influence RNA stability, splicing, localization, and translation (21–23). Interestingly, m^6^A modifications can exhibit both antiviral and proviral effects, depending on the viral context (24). Recent studies have shown that PRRSV infection alters the host’s epitranscriptome (25). For instance, PRRSV non-structural protein 9 (nsp9) enhances m^6^A modification and interleukin-13 (IL-13) expression by downregulating FTO (26). Nevertheless, the role of METTL3-mediated m^6^A modifications during PRRSV infection remains unclear.

In this study, we demonstrate that PRRSV infection modifies cellular m^6^A patterns, upregulates *METTL3* expression, and alters its cellular localization. METTL3 enhances PRRSV replication by inhibiting antiviral innate immunity. Mechanistically, METTL3 promotes autophagy by increasing m^6^A modification and transcription of *SQSTM1*, thereby facilitating the degradation of IKKε. These findings unveil a novel mechanism through which METTL3 promotes PRRSV infection by suppressing IFN-I production, highlighting potential antiviral targets for therapeutic intervention.

## RESULTS

### PRRSV RNA is m^6^A modified, and viral infection reprograms the host m^6^A epitranscriptome

**Fig 1:**
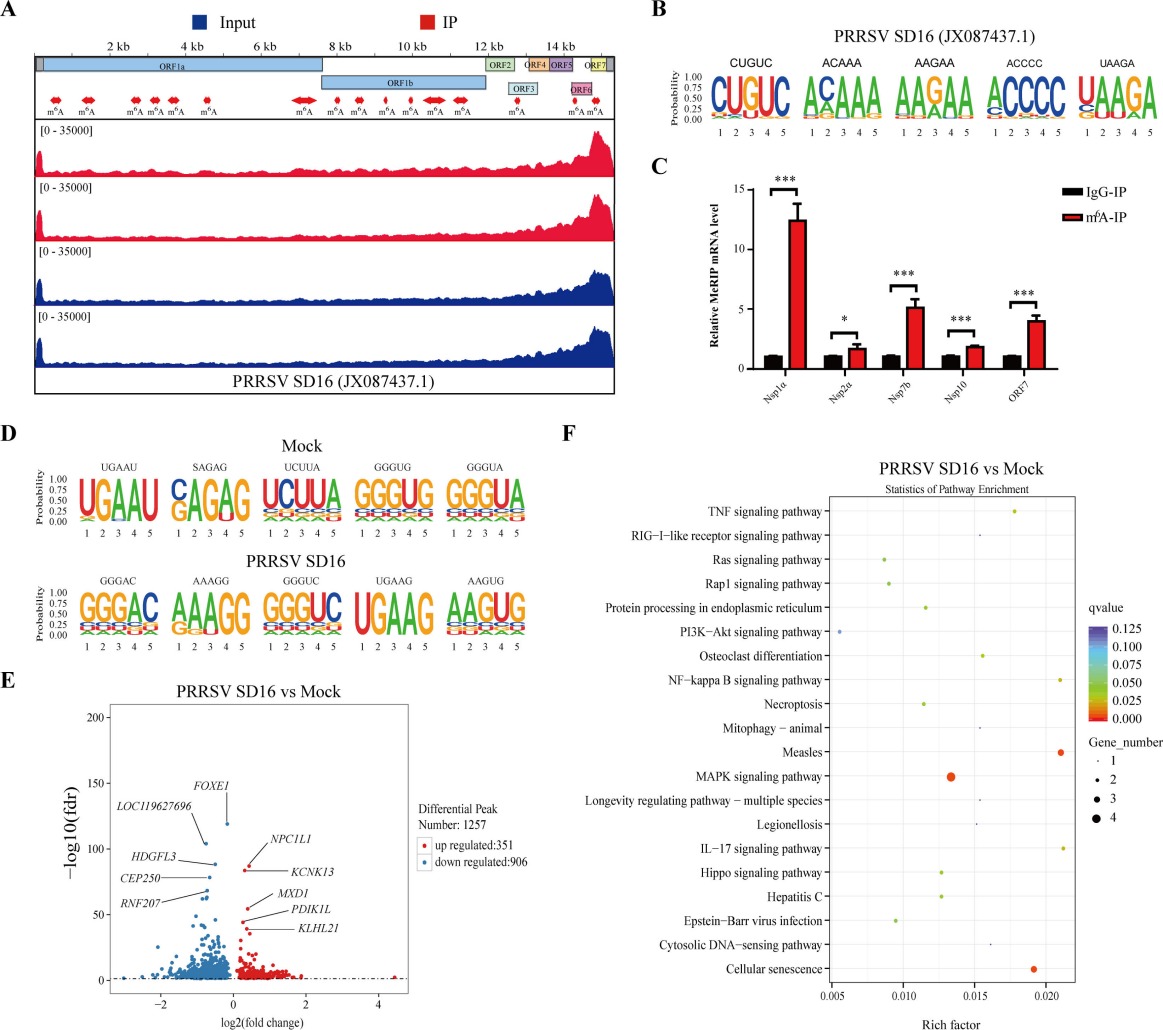
PRRSV RNA is m^6^A modified, and viral infection reprograms host m^6^A epitranscriptome. (**A**) Genome-wide distribution of m^6^A peaks across the PRRSV SD16 genome visualized using Integrative Genomics Viewer (IGV). (**B**) Consensus m^6^A methylation motifs identified in PRRSV genomic RNA during *in vitro* infection. (**C**) Validation of representative m^6^A peaks in *nsp1a*, *nsp2a*, *nsp7b*, *nsp10*, and *ORF7* by MeRIP-qPCR. (**D**) Comparison of enriched m^6^A motifs between PRRSV-infected and uninfected MARC-145 cells. (**E**) Volcano plot depicting peaks with significantly altered m^6^A modification levels in PRRSV-infected versus untreated MARC-145 cells. (**F**) Heat map showing significantly enriched pathways in PRRSV-infected cells, sorted by log_2_-transformed *P*-values.

To determine whether PRRSV RNA undergoes m^6^A modification, we extracted total RNA from large-scale cultures of PRRSV-infected MARC-145 cells and performed methylated RNA immunoprecipitation (MeRIP) using m^6^A-specific antibodies. Our analysis revealed multiple m^6^A peaks distributed throughout the PRRSV genome (Fig. 1A), with notable enrichment in the coding regions of ORF1a (seven sites), ORF1b (six sites), and ORFs 3, 6, and 7 (one site each). These m^6^A modifications predominantly occurred within the conserved RRACH motif (R = G or A; H = A, C, or U) (27), and their distribution was consistent with the canonical consensus sequence (Fig. 1B). MeRIP-qPCR further validated m^6^A enrichment in specific PRRSV genes, including *nsp1a*, *nsp2a*, *nsp7b*, *nsp10*, and *ORF7*, with *nsp1a* exhibiting the highest level of modification (Fig. 1C). These results demonstrate that PRRSV genomic RNA is subject to m^6^A modification during infection.

We next investigated whether PRRSV SD16 infection alters m^6^A modification patterns in host transcripts. MeRIP-seq analysis revealed a substantial shift in m^6^A motif distribution in cellular RNAs upon infection (Fig. 1D). Comparative analysis identified 351 genes with increased m^6^A modification levels and 906 genes with decreased modifications post-infection (Fig. 1E). Pathway enrichment analysis of these differentially modified genes identified 20 significantly affected signaling pathways, including tight junction regulation, spliceosome function, RNA transport, ribosome biogenesis, Ras/Rap1 signaling, endoplasmic reticulum protein processing, PI3K-Akt signaling, phospholipase D signaling, cancer pathways, oocyte meiosis, mRNA surveillance, mitophagy, human papillomavirus infection, Hippo signaling, focal adhesion, endocytosis, EGFR tyrosine kinase inhibitor resistance, and apelin signaling (Fig. 1F). By integrating MeRIP-seq and RNA-seq data, we identified 25 genes that exhibited concurrent alterations in both m^6^A modification and mRNA expression levels. To validate these findings, we selected three representative genes and performed MeRIP-qPCR and reverse transcription-quantitative PCR (RT-qPCR) analyses. *KITLG* (KIT ligand), a key regulator in the MAPK signaling pathway, displayed a coordinated decrease in both m^6^A methylation and transcript abundance (Fig. S1A through C). In contrast, the transcription factor *JUND* (Jun D proto-oncogene) showed reduced m^6^A modification but increased mRNA expression (Fig. S1D through F), suggesting a potential inverse relationship between m^6^A methylation and transcript stability. Notably, *EPHA2*, a receptor tyrosine kinase implicated in cell signaling, exhibited significant increases in both m^6^A modification and transcript expression (Fig. S1G through I). These results underscore the dynamic nature of m^6^A methylation during PRRSV infection and highlight its potential role in fine-tuning gene expression and host cellular responses.

### METTL3 positively regulates PRRSV infection

**Fig 2:**
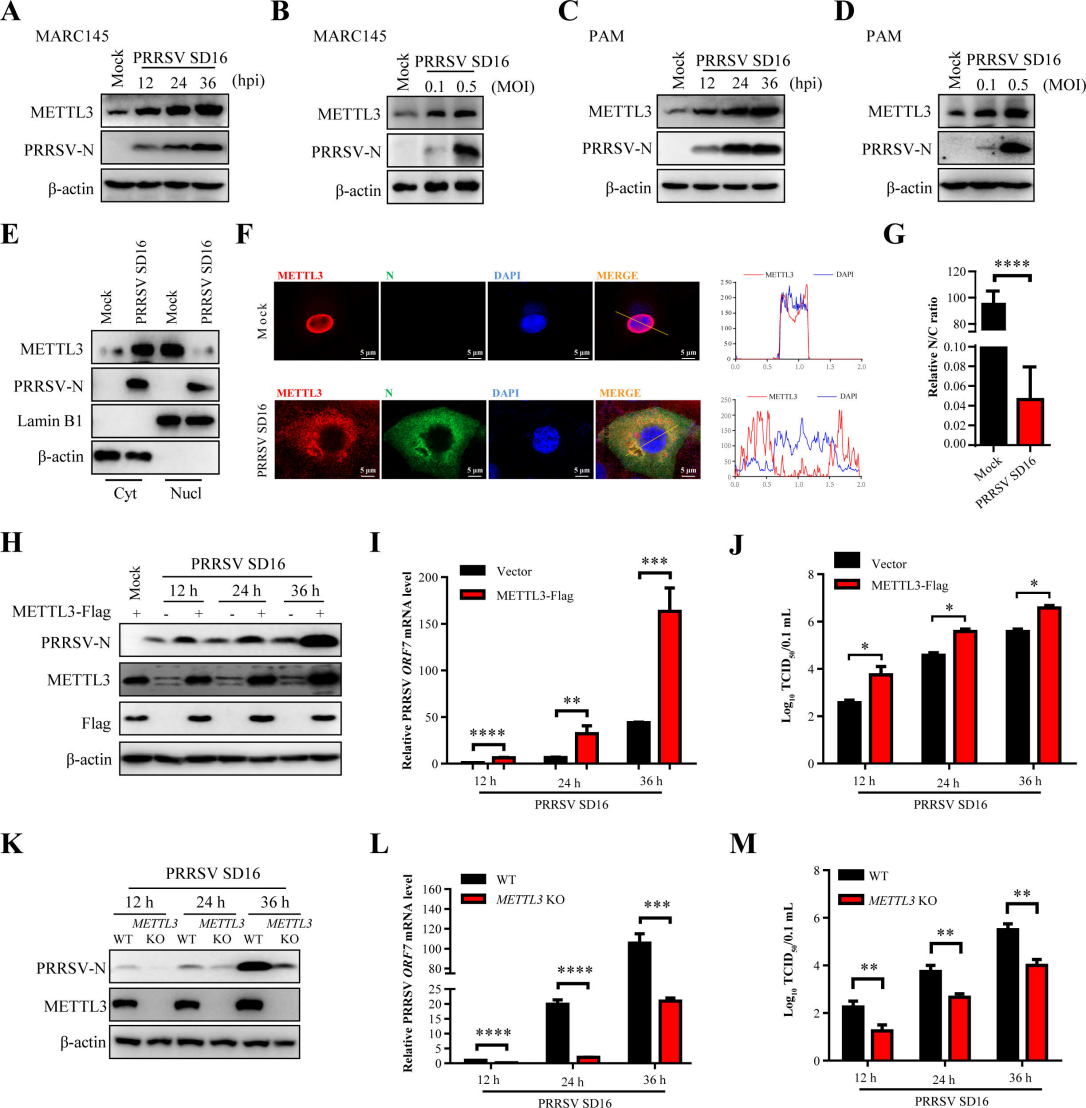
METTL3 positively regulates PRRSV infection. (**A**) Western blot analysis of METTL3 and PRRSV-N expression in MARC-145 cells infected with PRRSV at an MOI of 0.1 or 0.5, collected at 36 h. (**B**) Time-course analysis of METTL3 and PRRSV-N protein expression in MARC-145 cells infected with PRRSV at an MOI of 0.5, collected at 12, 24, and 36 h. (**C**) Time-course analysis of METTL3 and PRRSV-N protein expression in PAMs infected with PRRSV at an MOI of 0.5, collected at 12, 24, and 36 h. (**D**) Western blot analysis of METTL3 and PRRSV-N protein expression in PAMs infected with PRRSV at an MOI of 0.1 or 0.5, collected at 36 h. β-actin served as the loading control. (**E**) Subcellular localization of METTL3 in MARC-145 cells infected with PRRSV (MOI = 0.5) and collected at 36 h. Western blot was used to examine METTL3, PRRSV-N, nuclear Lamin B1, and cytoplasmic β-actin protein levels. (**F**) Immunofluorescence analysis of METTL3 and PRRSV-N subcellular localization in PRRSV-infected MARC-145 cells (MOI = 0.5) at 36 h. Nuclei were counterstained with DAPI. Confocal laser scanning microscopy was used to capture fluorescent images. (**G**) Quantification of METTL3 subcellular distribution in at least five cells using ImageJ. The nuclear-to-cytoplasmic (N/C) fluorescence intensity ratio was calculated and normalized to uninfected control cells to determine fold changes in localization. (H–J) MARC-145 cells were transfected with METTL3-Flag or an empty vector for 24 h, followed by PRRSV infection (MOI = 0.5). At 12, 24, and 36 h, cells were analyzed by western blot to detect METTL3 and PRRSV-N protein expression (H), RT-qPCR to measure PRRSV *ORF7* gene expression (fold changes normalized to *β-actin*) (**I**), and TCID_50_ assay to determine viral titers (**J**). (K–M) MARC-145 WT and *METTL3* KO cells were infected with PRRSV (MOI = 0.5). Cells were collected at 12, 24, and 36 h and analyzed using western blot (**K**), RT-qPCR (**L**), and TCID_50_ assay (**M**).

METTL3, the catalytic subunit of the m^6^A methyltransferase complex, is responsible for depositing over 90% of cellular m^6^A modifications (15). Given the pronounced remodeling of the host m^6^A landscape during PRRSV infection observed in our MeRIP-seq data, we further investigated how PRRSV affects METTL3 expression and its functional involvement in viral replication. We first examined METTL3 protein expression in PRRSV-infected MARC-145 cells. MARC-145 cells were infected with PRRSV, and METTL3 protein levels were assessed via western blot at 12, 24, and 36 h post-infection. The results demonstrated a time-dependent increase in METTL3 protein expression during PRRSV infection (Fig. 2A). To determine whether this upregulation was dose-dependent, MARC-145 cells were infected at different multiplicities of infection (MOI) of 0.1 and 0.5. Compared to cells infected with an MOI of 0.1, cells infected with an MOI of 0.5 exhibited significantly higher METTL3 expression (Fig. 2B). Similar trends were observed in porcine alveolar macrophages (PAMs), where METTL3 expression increased in both time-dependent and dose-dependent manners (Fig. 2C and D). These results suggest that the induction of METTL3 is directly linked to PRRSV replication. Next, we investigated the effect of PRRSV infection on the subcellular localization of METTL3. Nuclear and cytoplasmic fractionation assays revealed that PRRSV infection promoted the translocation of METTL3 from the nucleus to the cytoplasm. Specifically, compared to uninfected cells, PRRSV-infected MARC-145 cells displayed increased METTL3 levels in the cytoplasm and decreased levels in the nucleus (Fig. 2E). This observation was further corroborated by immunofluorescence assay (IFA) (Fig. 2F). Quantitative analysis of the nuclear-to-cytoplasmic ratio of METTL3 fluorescence signals across multiple cells (*N* ≥ 5) revealed that PRRSV infection markedly altered the subcellular distribution of METTL3, promoting its translocation from the nucleus to the cytoplasm (Fig. 2G). All these results suggested that the expression pattern of METTL3 was altered during PRRSV infection.

To validate the role of METTL3 in PRRSV infection, METTL3-Flag was transfected into MARC-145 cells. After 24 h, cells were infected with PRRSV, and samples were collected at 12, 24, and 36 h for analysis via western blot, RT-qPCR, and 50% tissue culture infective dose (TCID_50_) assays. The ectopic expression of METTL3 significantly increased the expression of the PRRSV N protein (Fig. 2H), the mRNA levels of *ORF7* (Fig. 2I), and viral titers (Fig. 2J) at all examined time points. Conversely, to evaluate the effect of endogenous METTL3 on PRRSV replication, *METTL3* knockout (KO) MARC-145 cells were generated using CRISPR-Cas9. Western blot, RT-qPCR, and TCID_50_ assays were conducted on PRRSV-infected cells collected at 12, 24, and 36 h. In contrast to the control group, the *METTL3* KO cells exhibited significantly reduced expression of the PRRSV N protein (Fig. 2K), decreased mRNA levels of *ORF7* (Fig. 2L), and lower viral titers (Fig. 2M). Together, these results demonstrate that METTL3 positively regulates PRRSV replication, highlighting its role as a key host factor facilitating viral proliferation.

### METTL3 suppresses the antiviral response

One of the hallmark features of PRRSV is its capacity to subvert host immunity (28). To investigate this aspect, we infected MARC-145 cells with PRRSV at MOIs of 0.1 and 0.5 for 36 h, followed by Poly I:C treatment for 12 h. The expression levels of *IFNB1* and downstream antiviral genes *ISG15* were then quantified using RT-qPCR. PRRSV infection significantly suppressed the transcription levels of *IFNB1* and *ISG15* induced by Poly I:C stimulation (Fig. S2A and B). Meanwhile, the replication of PRRSV was also inhibited (Fig. S2C). Furthermore, PRRSV infection substantially suppressed the phosphorylation of IRF3 (pIRF3) and the nuclear translocation of IRF3 in response to Poly I:C stimulation (Fig. S2D and E). These findings confirm that PRRSV possesses immunosuppressive properties (1, 10, 29).

**Fig 3:**
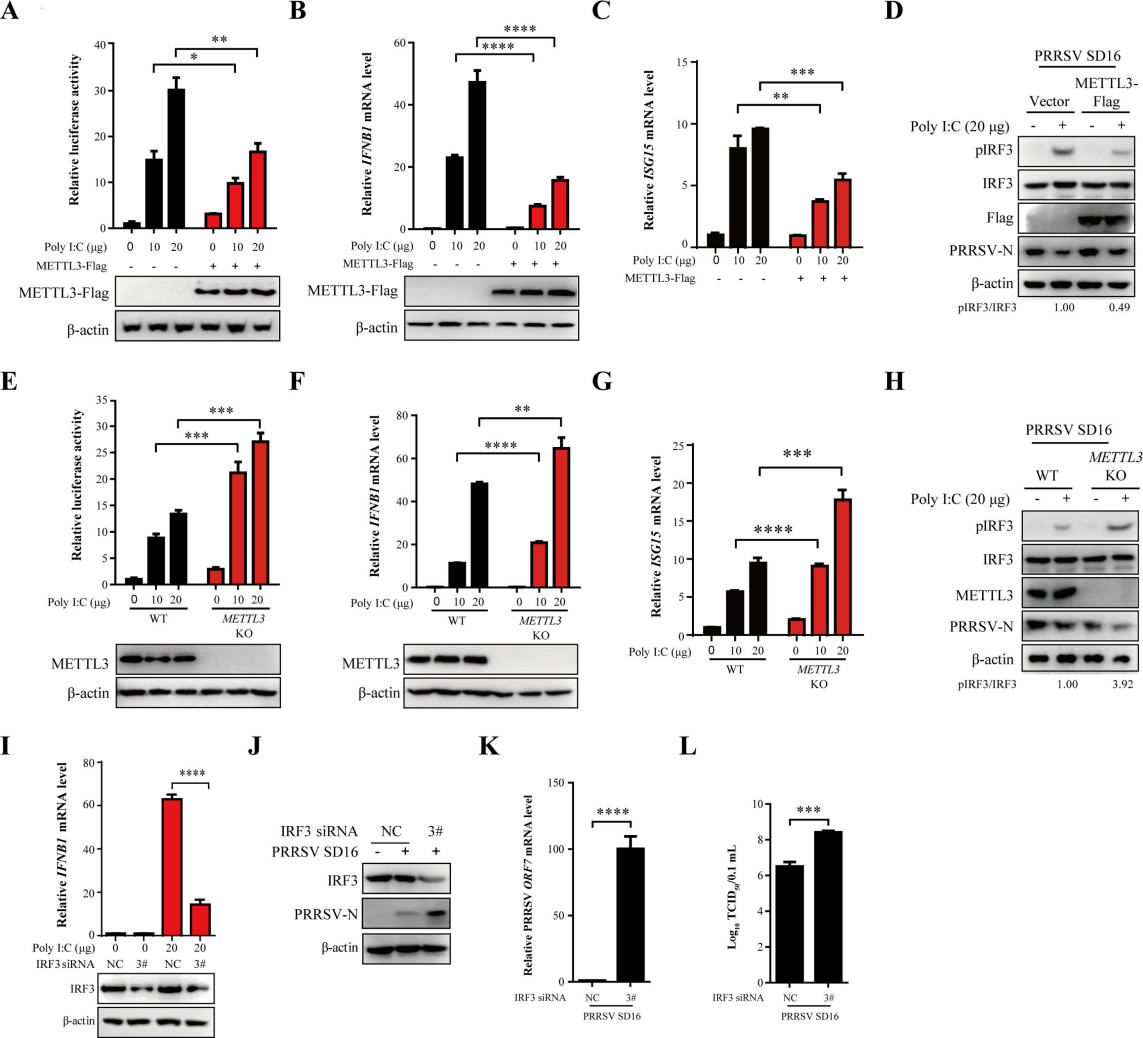
METTL3 suppresses the antiviral response. (**A**) Dual-luciferase assay in HEK293T cells co-transfected with IFNB-Luc (100 ng), pRL-TK (25 ng), and either METTL3-Flag (250 ng) or an empty plasmid (250 ng) for 12 h. Cells were subsequently treated with 10 or 20 µg/mL Poly I:C for 12 h. (**B and C**) RT-qPCR analysis of *IFNB1* (**B**) and *ISG15* (**C**) transcription (fold changes normalized to *β-actin*) in MARC-145 cells transfected with METTL3-Flag (2 µg) or an empty vector (2 µg) for 24 h, followed by treatment with 10 or 20 µg/mL Poly I:C for 12 h. (**D**) MARC-145 cells transfected with METTL3-Flag or an empty vector for 24 h were infected with PRRSV (MOI = 0.5). At 24 h, cells were treated with 20 µg/mL Poly I:C for 12 h. Western blot analysis was performed to detect pIRF3, IRF3, METTL3, and PRRSV-N protein levels. (**E**) Dual-luciferase assay in MARC-145 WT and *METTL3* KO cells co-transfected with IFNB-Luc (100 ng), pRL-TK (25 ng), and METTL3-Flag (100, 200, and 250 ng). (**F and G**) MARC-145 WT and *METTL3* KO with 20 µg/mL Poly I:C for 12 h. Western blot analysis was used to detect METTL3 expression, and RT-qPCR was conducted to evaluate *IFNB1* (**F**) and *ISG15* (**G**) transcription (fold changes normalized to *β-actin*). (**H**) Western blot analysis of pIRF3, IRF3, METTL3, and PRRSV-N protein levels in MARC-145 WT and *METTL3* KO cells infected with PRRSV (MOI = 0.5) for 24 h, followed by treatment with 20 µg/mL Poly I:C for 12 h. (**I**) RT-qPCR analysis of *IFNB1* transcription (fold changes normalized to *β-actin*) in MARC-145 cells transfected with small interfering RNA (siRNA) targeting *IRF3* following treatment with 20 µg/mL Poly I:C. (J–L) MARC-145 cells were transfected with *IRF3* siRNA or negative control siRNA (NC) for 24 h, followed by infection with PRRSV (MOI = 0.5). Cells were analyzed at 36 h using western blot (**J**), RT-qPCR (**K**), and TCID_50_ assay (**L**).

To elucidate the molecular mechanism underlying METTL3-mediated enhancement of PRRSV replication, we systematically investigated its effects on the expression of IFN-I. First, HEK293T cells were co-transfected with METTL3-Flag, a firefly luciferase reporter plasmid for the *IFNB1* promoter (IFNB-Luc), and the Renilla luciferase plasmid (pRL-TK) as an internal control. The cells were then stimulated with Poly I:C (10 or 20 µg/mL) for 12 h and assessed using a dual-luciferase reporter assay. Compared to cells transfected with an empty vector, METTL3 overexpression significantly suppressed *IFNB1* promoter activity induced by Poly I:C (Fig. 3A). Similarly, METTL3-Flag was transfected into MARC-145 cells, followed by Poly I:C stimulation (10 or 20 µg/mL). RT-qPCR analysis showed that METTL3 overexpression markedly reduced *IFNB1* and *ISG15* mRNA expression compared to the control group (Fig. 3B and C). MARC-145 cells were transfected with METTL3-Flag or an empty vector for 24 h, followed by PRRSV infection and stimulation with 20 µg/mL Poly I:C for 12 h. Western blot analysis revealed that, compared to the control group, overexpression of METTL3 significantly suppressed the phosphorylation of IRF3 (Fig. 3D). Further analysis in HEK293T cells transfected with varying doses of METTL3-Flag demonstrated a dose-dependent suppression of *IFNB1* promoter activity (Fig. S3A), transcription levels (Fig. S3B), and *ISG15* mRNA expression levels (Fig. S3C) in response to Poly I:C stimulation. Conversely, Poly I:C-induced *IFNB1* promoter activity (Fig. 3E), transcription levels (Fig. 3F), and *ISG15* mRNA expression levels (Fig. 3G) were significantly higher in *METTL3* KO cells compared to WT cells. Similarly, in *METTL3* KO MARC-145 cells, the loss of METTL3 significantly upregulated the phosphorylation of IRF3 in response to Poly I:C stimulation (Fig. 3H). Collectively, these results indicate that METTL3 negatively regulates *IFNB1* production.

IRF3 functions as an upstream regulator of *IFNB1* in innate immunity (30). The antiviral function of IRF3 is well established. To assess its role in IFNB1 transcription, we first examined IFNB1 expression following IRF3 knockdown. The results showed that silencing IRF3 significantly reduced Poly I:C-induced *IFNB1* transcription (Fig. 3I). Western blot (Fig. 3J), RT-qPCR (Fig. 3K), and viral titer (Fig. 3L) analyses consistently demonstrated that interference with *IRF3* significantly enhanced PRRSV replication.

### METTL3 interacts with TBK1/IKKε

**Fig 4:**
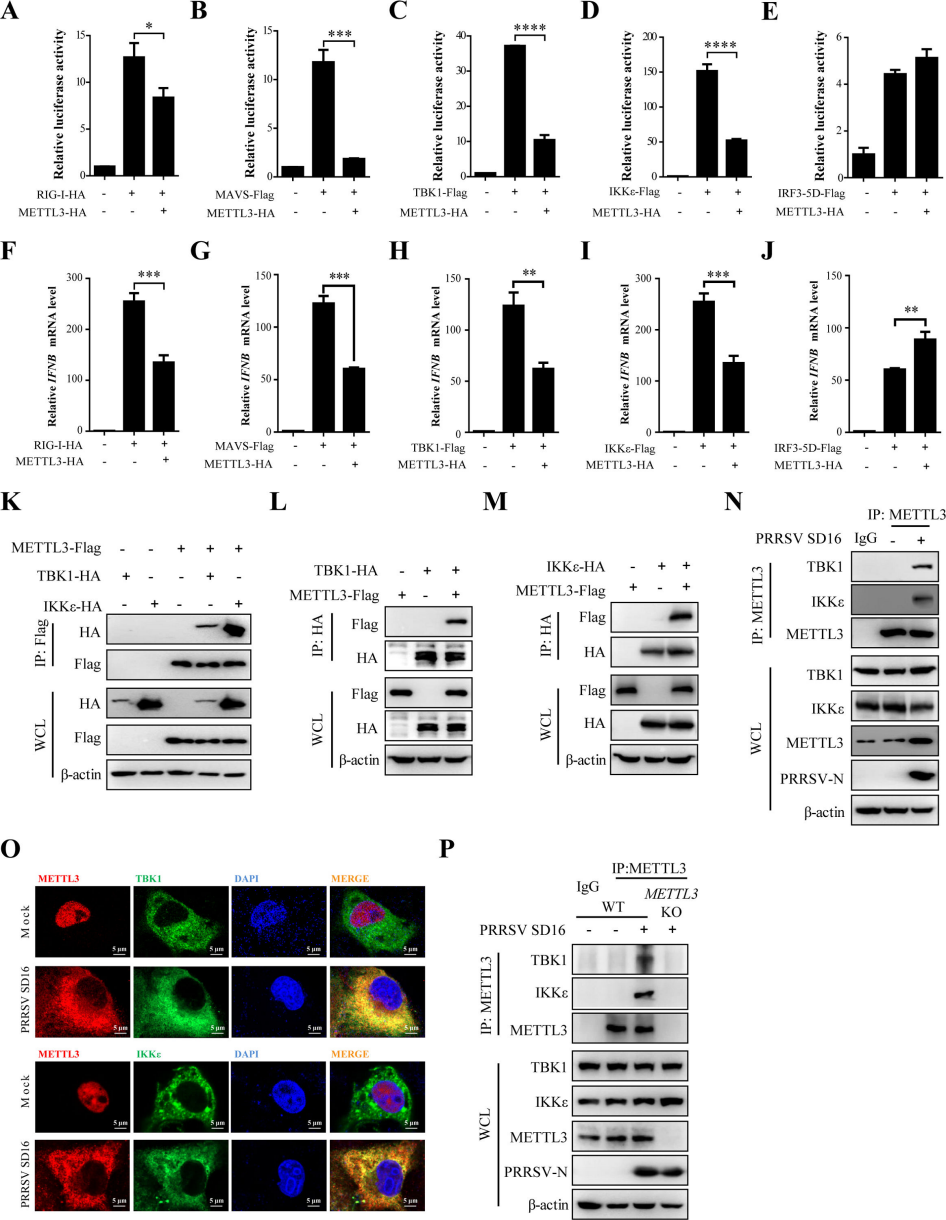
METTL3 interacts with TBK1/IKKε. (**A–E**) HEK293T cells were co-transfected with IFNB-Luc, pRL-TK, and HA-METTL3, along with constructs or empty vectors expressing RIG-I-HA (**A**), MAVS-Flag (**B**), TBK1-Flag (**C**), IKKε-Flag (**D**), or IRF3-5D-Flag (**E**). After 24 h, cells were harvested, and a dual-luciferase assay was performed. (**F–J**) MARC-145 cells were co-transfected with HA-METTL3, as well as construct plasmids or empty vectors expressing RIG-I-HA (**F**), MAVS-Flag (**G**), TBK1-Flag (**H**), IKKε-Flag (**I**), and IRF3-5D-Flag (**J**). Twenty-four hours after transfection, cells were harvested for protein expression analysis via western blot, with HA and Flag antibodies used to probe the blots. Additionally, the transcription of *IFNB1* was quantified via RT-qPCR (fold changes normalized to *β-actin*). (**K**) HEK293T cells co-transfected with METTL3-Flag and either TBK1-HA or IKKε-HA were lysed 48 h later. Cell lysates were immunoprecipitated with an anti-Flag antibody. Whole-cell lysates (WCL) and co-immunoprecipitation (co-IP) complexes were analyzed by western blot using anti-Flag, anti-HA, and anti-β-actin antibodies. (**L and M**) HEK293T cells were transfected with TBK1-HA (**L**) or IKKε-HA (**M**) along with METTL3-Flag. At 24 h, cell lysates were immunoprecipitated with an anti-HA antibody and analyzed by western blot with the specified antibodies. (**N**) MARC-145 cells infected with PRRSV (MOI = 0.5) for 36 h were lysed, and METTL3 was immunoprecipitated. Western blot was used to analyze interacting proteins. (**O**) Immunofluorescence microscopy showing the subcellular localization of METTL3 and TBK1 or IKKε in PRRSV-infected MARC-145 cells (MOI = 0.5) at 36 h. Nuclei were counterstained with DAPI. Confocal laser scanning microscopy was used to capture images. (**P**) MARC-145 WT and *METTL3* KO cells were infected with PRRSV (MOI = 0.5) for 36 h. METTL3 was immunoprecipitated, and interacting proteins were detected by western blot.

Given these findings, we next examined the impact of METTL3 overexpression on *IFNB1* activation across various components of the RIG-I signaling pathway. The results revealed that METTL3 overexpression inhibited RIG-I, MAVS, TBK1, and IKKε-mediated activation of *IFNB1,* but did not affect IRF3-mediated activation. Dual-luciferase reporter assay results (Fig. 4A through E) aligned with RT-qPCR data (Fig. 4F through J).

To identify METTL3’s precise targets, HEK293T cells were co-transfected with METTL3-Flag and HA-tagged TBK1 or IKKε expression constructs, followed by co-immunoprecipitation (co-IP) assays. Both TBK1 and IKKε proteins were detected in immune complexes precipitated with anti-Flag antibody (Fig. 4K). In reciprocal co-IP experiments, METTL3 was also efficiently co-immunoprecipitated with TBK1 and IKKε proteins using anti-HA antibody (Fig. 4L and M). Interestingly, no interaction between endogenous METTL3 and TBK1 or IKKε was observed in uninfected MARC-145 cells, but these associations were evident following PRRSV infection (Fig. 4N). Subsequently, we assessed the subcellular localization of METTL3 with TBK1 and IKKε proteins. IFA results showed partial co-localization of METTL3 with TBK1 and IKKε in the cytoplasm of PRRSV-infected cells (Fig. 4O). In *METTL3* KO cells, the interaction between METTL3 and TBK1 or IKKε observed during PRRSV infection was abolished (Fig. 4P). These results indicate that PRRSV infection leads to METTL3 targeting TBK1/IKKε.

### METTL3 suppresses IKKε protein expression, thereby enhancing PRRSV replication

**Fig 5:**
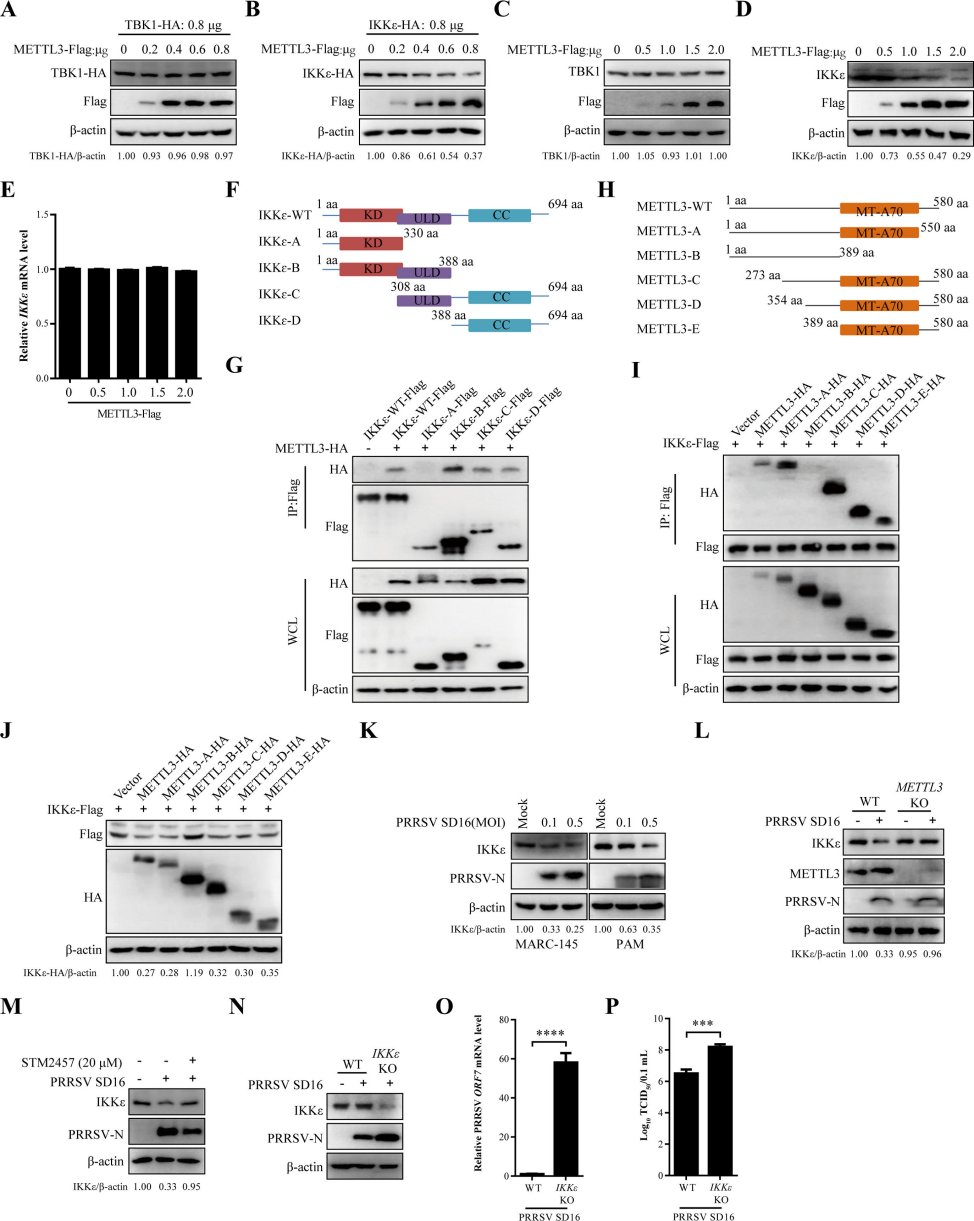
METTL3 suppresses IKKε protein expression, thereby enhancing PRRSV replication. (**A–D**) HEK293T or MARC-145 cells were transfected with varying amounts of METTL3-Flag or an empty plasmid. After 24 h, cells were harvested, and western blot analysis was conducted to detect TBK1 (**A and C**) and IKKε (**B and D**) protein expression. (**E**) RT-qPCR analysis of *IKKε* transcription in MARC-145 cells transfected with METTL3-Flag or an empty plasmid for 24 h. (**F**) Schematic representation of Flag-tagged truncated IKKε constructs. (**G**) HEK293T cells were co-transfected with IKKε-Flag (or its truncated constructs) and METTL3-HA. After 24 h, co-IP was performed to analyze protein interactions. (**H**) Schematic representation of HA-tagged truncated METTL3 constructs. (**I**) HEK293T cells were co-transfected with METTL3-HA (or its truncated constructs) and IKKε-Flag. After 24 h, co-IP was performed to analyze protein interactions. (**J**) Western blot analysis of IKKε protein expression in HEK293T cells co-transfected with METTL3-HA (or its mutants) and IKKε-Flag for 24 h. (**K**) PRRSV-infected MARC-145 cells or PAMs (MOI = 0.1 or 0.5) were harvested, and IKKε protein levels were analyzed by western blot. (**L**) Western blot analysis of IKKε, METTL3, and PRRSV-N protein expression in MARC-145 WT and *METTL3* KO cells infected with PRRSV (MOI = 0.5) for 36 h. (**M**) MARC-145 cells infected with PRRSV were treated with STM2457 or DMSO. Western blot analysis of IKKε and PRRSV-N protein expression. (**N–P**) Loss of IKKε enhances PRRSV replication. MARC-145 WT and *IKKε* KO cells were infected with PRRSV (MOI = 0.5) for 36 h. Western blot (**N**), RT-qPCR (**O**), and TCID_50_ assay (**P**) were performed to evaluate viral replication.

Given that METTL3 inhibits *IFNB1* expression and targets the TBK1/IKKε complex, we sought to analyze the regulatory relationship between METTL3 and TBK1/IKKε. To this end, TBK1-HA or IKKε-HA plasmids were transfected with varying concentrations of METTL3-Flag plasmid into HEK293T cells. After 24 h, western blot analysis revealed that the protein expression of TBK1 remained unaffected (Fig. 5A). However, the level of IKKε was inhibited by METTL3-Flag in a dose-dependent manner (Fig. 5B). Similarly, in MARC-145 cells, overexpression of METTL3 did not affect endogenous TBK1 levels (Fig. 5C), whereas IKKε protein levels decreased with increased METTL3 expression (Fig. 5D). We next evaluated *IKKε* mRNA levels. The findings indicated that *IKKε* transcript levels remained unchanged following transfection with increasing concentrations of METTL3-Flag (Fig. 5E). To identify the regions of IKKε and METTL3 responsible for their interaction and the inhibition of IKKε protein stability, a series of IKKε and METTL3 constructs with intact or truncated domains were generated (Fig. 5F and H). The results demonstrated that IKKε with only the N-terminal KD domain (IKKε-A-Flag) cannot interact with METTL3 (Fig. 5G). The METTL3 construct lacking the MT-A70 domain (METTL3-B-HA) was unable to interact with IKKε (Fig. 5I) and could not downregulate IKKε protein expression (Fig. 5J). These findings suggest that METTL3 specifically reduces IKKε protein expression via its MT-A70 domain.

To further investigate why METTL3 selectively affects IKKε but not TBK1 stability, we mapped the interaction domains between METTL3 and TBK1 (Fig. S4A). The results revealed that the C-terminal domain of TBK1 (TBK1-D-Flag) failed to interact with METTL3 (Fig. S4B). Additionally, a METTL3 construct lacking the MT-A70 domain (METTL3-B-HA) was unable to bind TBK1 (Fig. S4C), indicating the necessity of this domain for the interaction. We also assessed whether the enzymatic activity of METTL3 influences its interaction with the TBK1/IKKε complex. Notably, the catalytic site mutant METTL3-D395A-HA retained the ability to interact with both TBK1 and IKKε, similar to the wild-type METTL3-HA (Fig. S4D).

In both MARC-145 cells and PAMs, PRRSV infection significantly suppressed IKKε protein expression as MOI increased (Fig. 5K). Additionally, PRRSV failed to induce further degradation of IKKε in *METTL3* KO MARC-145 cells (Fig. 5L). The METTL3 catalytic activity inhibitor STM2457 was selected for additional investigation (31). Cytotoxicity assays using CCK-8 revealed that STM2457 concentrations of 10 and 20 µM were optimal for subsequent experiments (Fig. S5A). Dot blot analysis demonstrated that STM2457 inhibited total intracellular m^6^A modification, with 20 µM showing stronger effects (Fig. S5B). To evaluate the impact of STM2457 on PRRSV replication, western blot results indicated that STM2457 treatment inhibited PRRSV N protein expression in a concentration-dependent manner (Fig. S5C). Additionally, RT-qPCR analysis of PRRSV *ORF7* mRNA levels and TCID_50_ assays both confirmed the inhibitory effects of STM2457 on viral replication (Fig. S5D and E). We further assessed the regulatory effect of STM2457 on IKKε degradation during PRRSV infection. Western blot analysis showed that STM2457 treatment suppressed PRRSV-induced degradation of IKKε (Fig. 5M).

Finally, we evaluated the role of IKKε in PRRSV replication. Western blot results showed that *IKKε* KO promoted PRRSV N protein expression (Fig. 5N), while RT-qPCR analysis revealed increased PRRSV *ORF7* transcription (Fig. 5O). The replication capacity of progeny viruses was significantly elevated in *IKKε* KO cells (Fig. 5P). These findings indicate that METTL3 targets IKKε, disrupting their interaction with IRF3. Moreover, IKKε acts as a negative regulator of PRRSV replication, and its absence enhances viral replication.

### Autophagy mediates METTL3-dependent regulation of IKKε protein levels

**Fig 6:**
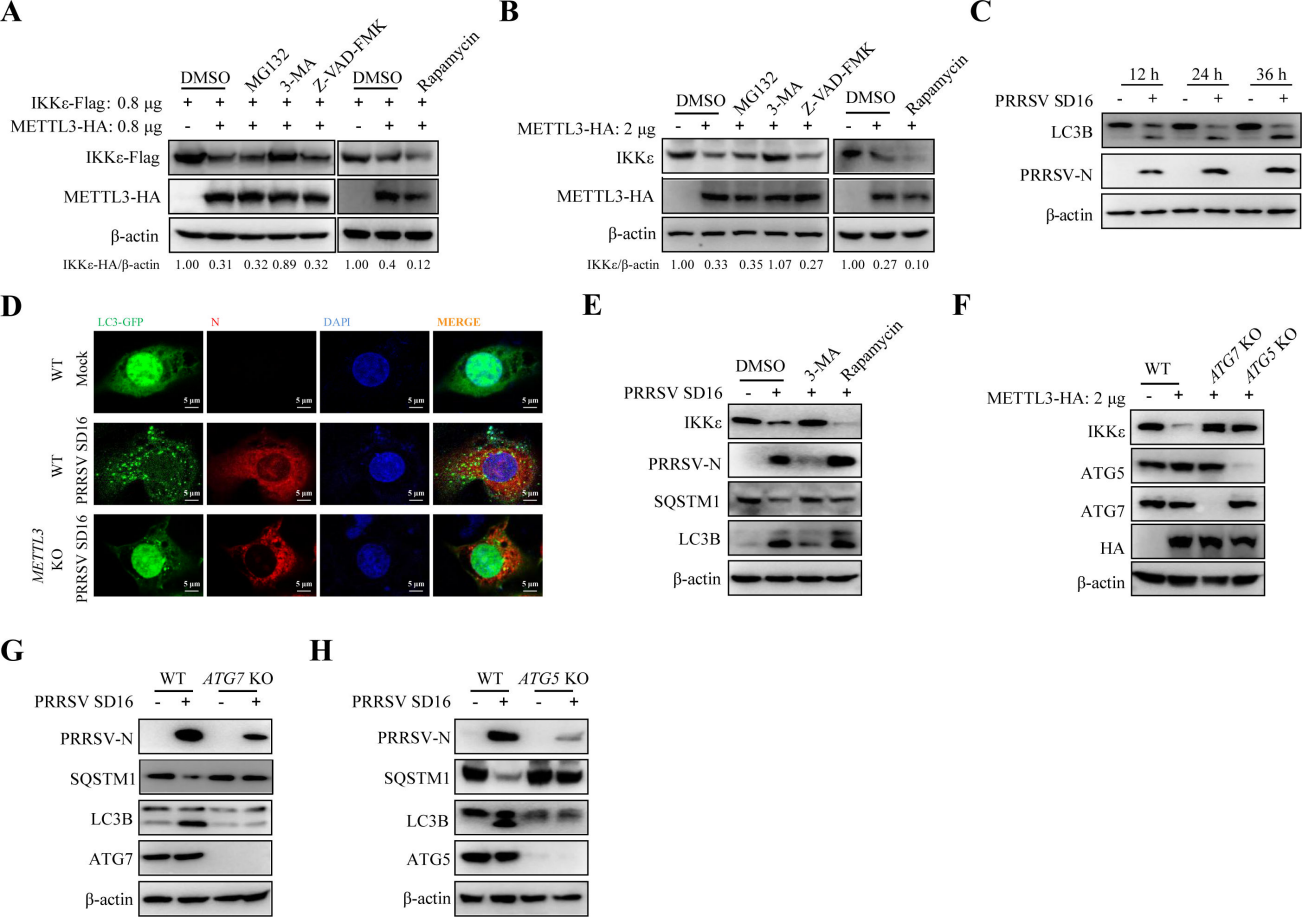
Autophagy mediates METTL3-dependent regulation of IKKε protein levels. (**A**) HEK293T cells co-transfected with METTL3-Flag and IKKε-HA were treated with MG132 (10 µM), 3-MA (10 mM), Z-VAD-FMK (10 µM), or Rapamycin (1 nM) for 12 h. Western blot analysis was performed to detect IKKε-Flag protein expression. (**B**) MARC-145 cells transfected with METTL3-Flag or an empty plasmid were treated with MG132, 3-MA, Z-VAD-FMK, or Rapamycin for 12 h. Western blot was conducted to analyze IKKε protein level. (**C**) Western blot analysis of LC3B and PRRSV-N expression in MARC-145 cells infected with PRRSV. (**D**) Immunofluorescence microscopy showing reduced LC3-GFP aggregation in *METTL3* KO cells during PRRSV infection. Fluorescence images were acquired by confocal laser scanning microscopy. (**E**) MARC-145 cells infected with PRRSV (MOI = 0.5) were treated with DMSO, 3-MA, or Rapamycin for 12 h. Western blot analysis was performed to detect IKKε, PRRSV-N, SQSTM1, and LC3B protein levels. (**F**) MARC-145 WT, *ATG7* KO, and *ATG5* KO cells were transfected with METTL3-HA and harvested 36 h later. Western blot analysis was conducted to detect IKKε, ATG7, ATG5, SQSTM1, and METTL3 protein expression. (**G and H**) MARC-145 WT, *ATG7* KO (**G**), and *ATG5* KO (**H**) cells were infected with PRRSV (MOI = 0.5) for 36 h. Western blot analysis was conducted to detect PRRSV-N, SQSTM1, LC3B, ATG7, and ATG5 protein expression.

The three principal intracellular degradation pathways—apoptosis, autophagy, and the ubiquitin-proteasome system—were investigated to determine how METTL3 mediates IKKε degradation (32). HEK293T cells were co-transfected with METTL3-Flag and IKKε-HA and treated with inhibitors for these pathways: MG132 (ubiquitin-proteasome pathway), 3-MA (autophagy pathway), or Z-VAD-FMK (apoptosis pathway). Western blot results indicated that treatment with 3-MA nearly completely restored IKKε protein levels, effectively reversing METTL3-induced degradation. In contrast, neither Z-VAD-FMK nor MG132 reversed IKKε degradation (Fig. 6A). Treatment with the autophagy inducer rapamycin exacerbated IKKε degradation mediated by METTL3. Validation in MARC-145 cells confirmed that 3-MA prevented endogenous IKKε degradation by METTL3 (Fig. 6B). PRRSV infection is known to induce autophagy in host cells (33). Our results also suggest that PRRSV infection leads to elevated METTL3 protein expression. We hypothesized that PRRSV induced IKKε degradation. MARC-145 cells infected with PRRSV showed increased LC3B expression in a time-dependent manner (Fig. 6C). IFA results showed marked aggregation of LC3-GFP in PRRSV-infected cells, which was diminished in METTL3-deficient cells (Fig. 6D). Furthermore, 3-MA inhibited PRRSV-induced LC3B generation, SQSTM1 degradation, and PRRSV-N protein expression. Conversely, rapamycin promoted autophagy and increased PRRSV-N protein levels (Fig. 6E). These findings collectively suggest that PRRSV-induced autophagy facilitates METTL3-mediated IKKε degradation.

To confirm this, we constructed MARC-145 knockout cells lacking autophagy genes *ATG7* and *ATG5* (referred to as *ATG7* KO and *ATG5* KO, respectively) (34). Overexpression of *METTL3* in *ATG7* KO or *ATG5* KO cells failed to induce IKKε degradation (Fig. 6F). Additionally, PRRSV-N protein expression was significantly reduced in *ATG7* KO and *ATG5* KO cells compared to WT MARC-145 cells (Fig. 6G and H). Together, these results demonstrate that METTL3 degrades IKKε via PRRSV-induced autophagy.

### METTL3 enhances the recognition of IKKε by the cargo receptor SQSTM1

**Fig 7:**
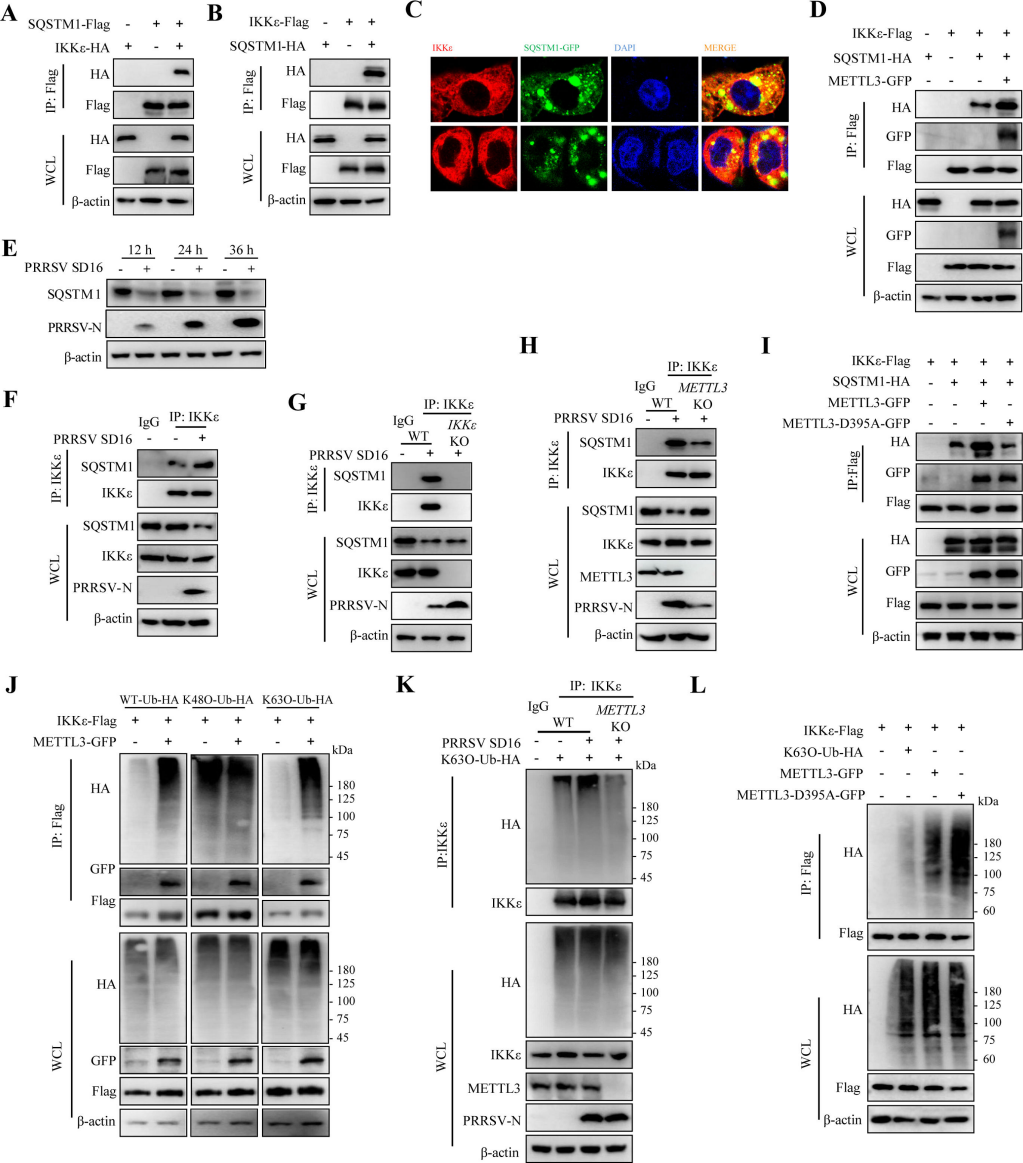
METTL3 enhances the recognition of IKKε by the cargo receptor SQSTM1. (**A**) Co-IP analysis of SQSTM1-Flag and IKKε-HA interaction. HEK293T cells were co-transfected with SQSTM1-Flag and IKKε-HA. After 24 h, cell lysates were immunoprecipitated with an anti-Flag antibody and analyzed by western blot with specified antibodies. (**B**) Reciprocal co-IP showing interaction between IKKε-Flag and SQSTM1-HA in HEK293T cells transfected with both plasmids. After 24 h, lysates were immunoprecipitated with an anti-Flag antibody and analyzed by western blot. (**C**) Immunofluorescence microscopy of PRRSV-infected MARC-145 cells (MOI = 0.5) at 36 h showing colocalization of SQSTM1-GFP and IKKε. Nuclei were counterstained with DAPI. Images were acquired using a confocal laser scanning microscope. (**D**) Co-IP analysis of IKKε-Flag, SQSTM1-HA, and METTL3-GFP interaction in HEK293T cells transfected with the indicated plasmids. After 24 h, lysates were immunoprecipitated with an anti-Flag antibody and analyzed by western blot. (**E**) Western blot analysis of SQSTM1 and PRRSV-N expression in MARC-145 cells infected with PRRSV. (**F**) Western blot analysis of PRRSV-infected MARC-145 cells (MOI = 0.5) at 36 h to detect IKKε and SQSTM1 interaction via co-IP. (**G**) Co-IP of PRRSV-infected MARC-145 WT and *IKKε* KO cells (MOI = 0.5) at 36 h to examine IKKε interaction with SQSTM1. (**H**) Western blot analysis of METTL3-dependent interaction between IKKε and SQSTM1 in PRRSV-infected MARC-145 WT and *METTL3* KO cells (MOI = 0.5) at 36 h. (**I**) Co-IP analysis of IKKε-Flag, SQSTM1-HA, METTL3-GFP, and METTL3-D395A-GFP interaction in HEK293T cells transfected with the indicated plasmids. After 24 h, lysates were immunoprecipitated with an anti-Flag antibody and analyzed by western blot. (**J**) HEK293T cells were co-transfected with WT-Ub-HA, K48O-Ub-HA, K63O-Ub-HA, IKKε-Flag, and METTL3-GFP plasmids. Co-IP was performed 24 h later to analyze ubiquitination patterns and *METTL3*-mediated effects. (**K**) MARC-145 WT and *METTL3* KO cells were transfected with K63O-Ub-HA plasmid, infected with PRRSV (MOI = 0.5), and harvested 36 h later. Co-IP with anti-IKKε antibody was performed to detect K63-linked ubiquitination. (**L**) HEK293T cells were co-transfected with METTL3-GFP, METTL3-D395A-HA in IKKε-Flag, K63O-Ub-HA plasmids, respectively. Twenty-four hours later, co-IP was performed to analyze the ubiquitination pattern.

SQSTM1 is a key autophagy cargo receptor that recognizes ubiquitinated proteins via its ubiquitin-associated domain (35). Previous studies have shown that SQSTM1 can recognize IKKε (36). Using exogenous co-IP assays, we confirmed the interaction between SQSTM1 and IKKε (Fig. 7A and B). IFA results further demonstrated cytoplasmic puncta, indicating discrete aggregates of IKKε with SQSTM1-GFP (Fig. 7C). To determine whether METTL3 regulates the interaction between SQSTM1 and IKKε, co-IP assays were performed, showing that METTL3 enhances the binding of SQSTM1 to IKKε (Fig. 7D). Next, we examined the protein level of SQSTM1 after PRRSV infection and found that the protein level of SQSTM1 gradually decreased with the prolongation of PRRSV infection (Fig. 7E). Importantly, endogenous IP assays revealed that PRRSV infection enhanced the interaction between IKKε and SQSTM1 (Fig. 7F). However, this interaction was absent in *IKKε* KO cells (Fig. 7G). Moreover, *METTL3* KO cell lines exhibited a marked reduction in the endogenous interaction between IKKε and SQSTM1 (Fig. 7H). These results demonstrate that METTL3 plays a pivotal role in enhancing the interaction between IKKε and the cargo receptor SQSTM1. To further investigate whether the methyltransferase activity of METTL3 is involved in the interaction between SQSTM1 and IKKε, we compared the effects of METTL3-GFP and the enzymatically inactive mutant METTL3-D395A-GFP. In contrast to METTL3-GFP, the METTL3-D395A-GFP mutant could not promote the interaction between IKKε and SQSTM1 (Fig. 7I).

Previous studies have shown that IKKε is subject to ubiquitin modification (37). To investigate changes in ubiquitination during IKKε autophagy degradation, HEK293T cells were co-transfected with IKKε-Flag along with WT-Ub-HA, K48O-Ub-HA, or K63O-Ub-HA plasmids. Through co-IP analysis, it was revealed that IKKε undergoes substantial ubiquitination, with K48-linked and K63-linked ubiquitin modifications occurring at comparable levels (Fig. S6). Next, we evaluated whether METTL3 influences IKKε degradation by modulating its ubiquitination. HEK293T cells were co-transfected with METTL3-GFP, IKKε-Flag, and ubiquitin plasmids. The results indicated that METTL3 significantly enhanced WT ubiquitination of IKKε, but did not affect K48-linked ubiquitination. However, METTL3 markedly increased K63-linked ubiquitination of IKKε (Fig. 7J). Furthermore, *METTL3* KO cell lines showed a significant reduction in K63-linked ubiquitination of IKKε (Fig. 7K). The METTL3-D395A-GFP mutant further increased the K63-linked ubiquitination of IKKε (Fig. 7L). These findings demonstrate that METTL3 orchestrates the SQSTM1-mediated recognition of IKKε through precise regulation of K63-linked polyubiquitination on IKKε. More critically, the methyltransferase activity of METTL3 is involved in this regulatory process.

### METTL3 regulates m^6^A modification of SQSTM1 and mediates autophagy degradation of IKKε

**Fig 8:**
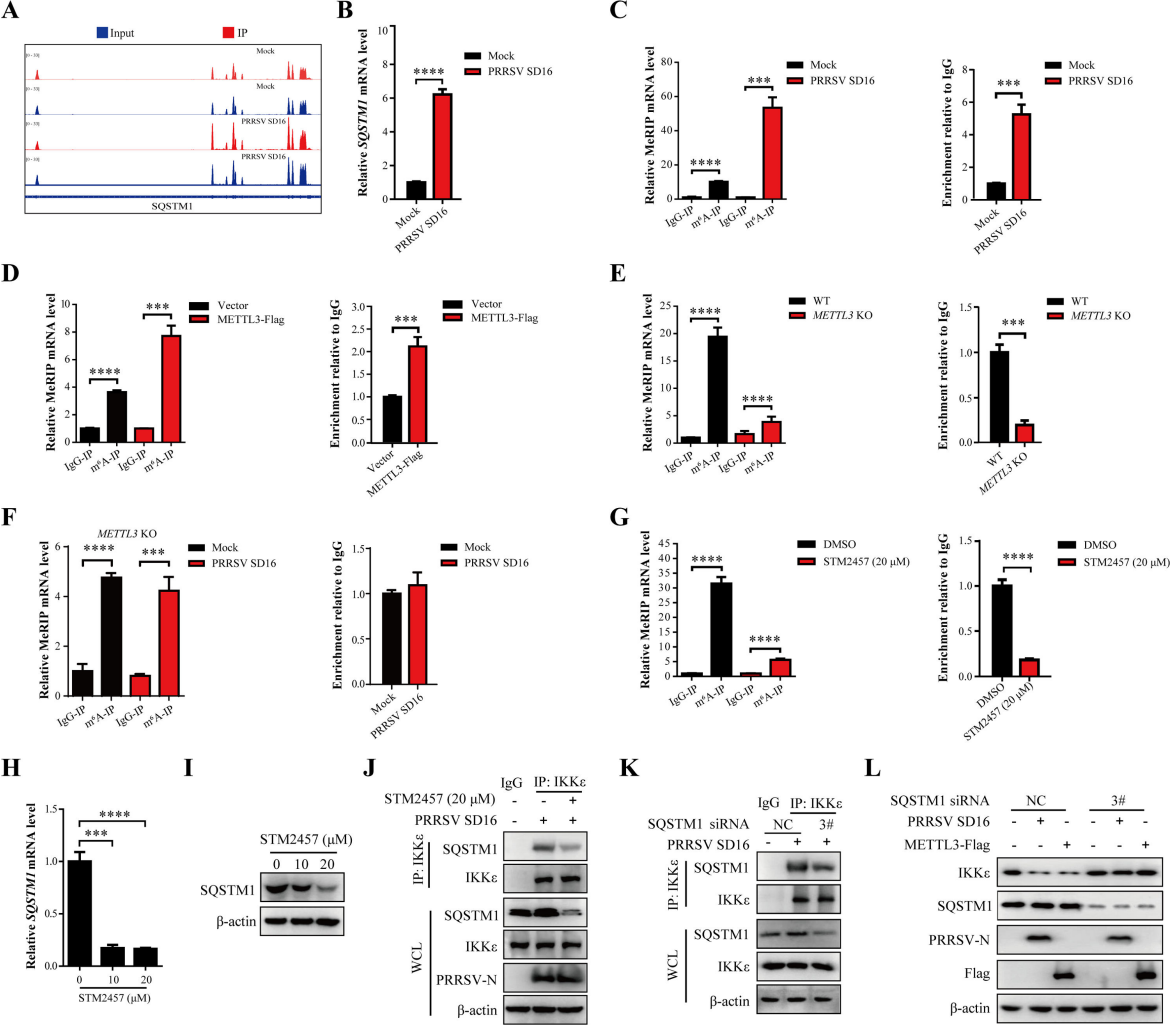
METTL3 regulates m^6^A modification of SQSTM1 and mediates autophagy degradation of IKKε. (**A**) Visualization of m^6^A peaks in SQSTM1 in PRRSV-infected or uninfected MARC-145 cells is shown by IGV. (**B**) RT-qPCR detection of *SQSTM1* mRNA expression (fold changes normalized to *β-actin*) in MARC-145 cells infected with PRRSV (MOI = 0.5) at 36 h. (**C**) Validation of m^6^A peaks in SQSTM1 in PRRSV-infected or uninfected MARC-145 cells is shown in panel (**A**) by MeRIP-qPCR. Relative enrichment of m^6^A was determined by calculating the fold change of IP to Input Ct values (IP/Input). (**D**) Overexpression of METTL3 in MARC-145 cells increases m^6^A modification of *SQSTM1* mRNA, as determined by MeRIP-qPCR. Relative enrichment of m^6^A was determined by calculating the fold change of IP to Input Ct values (IP/Input). (**E**) *METTL3* KO decreases *SQSTM1* m^6^A modification, as shown by MeRIP-qPCR of MARC-145 WT and *METTL3* KO cells. Relative enrichment of m^6^A was determined by calculating the fold change of IP to Input Ct values (IP/Input). (**F**) WT and *METTL3* KO cells were infected with PRRSV at an MOI of 0.5. At 36 h, total RNA was extracted from the cells and subjected to MeRIP-qPCR assays. Relative enrichment of m^6^A was determined by calculating the fold change of IP to Input Ct values (IP/Input). (**G**) STM2457 (20 µM) treatment inhibits m^6^A modification of *SQSTM1* mRNA in MARC-145 cells, as analyzed by MeRIP-qPCR. Relative enrichment of m^6^A was determined by calculating the fold change of IP to Input Ct values (IP/Input). (**H and I**) To evaluate the effect of STM2457 on SQSTM1 expression, MARC-145 cells were analyzed by RT-qPCR (fold changes normalized to *β-actin*) (H) and western blot (I). (**J**) STM2457 suppresses IKKε interaction with SQSTM1 in PRRSV-infected MARC-145 cells (MOI = 0.5). Lysates were immunoprecipitated with an anti-IKKε antibody and analyzed by western blot. (**K**) *SQSTM1* knockdown by siRNA decreases IKKε interaction in PRRSV-infected MARC-145 cells (MOI = 0.5), as analyzed by co-IP. (**L**) *SQSTM1* knockdown prevents METTL3-mediated IKKε degradation during PRRSV infection. Western blot analysis of Flag, SQSTM1, IKKε, and PRRSV-N protein expression.

Previous results indicated that the methylase activity of METTL3 appears to play a role in the interaction of SQSTM1 with IKKε, which we explored further. Integrated MeRIP-seq and RNA-seq analysis revealed that both m^6^A modification and the expression of SQSTM1 were elevated following PRRSV infection. The SQSTM1 transcript contains m^6^A modifications (Fig. 8A). The mRNA levels of *SQSTM1* were significantly elevated following PRRSV infection (Fig. 8B). MeRIP-qPCR analysis confirmed a marked upregulation of m^6^A modification on *SQSTM1* mRNA during PRRSV infection (Fig. 8C). Overexpression of METTL3 further enhanced the m^6^A modification level of SQSTM1 (Fig. 8D), whereas *METTL3* KO significantly reduced it (Fig. 8E), and even PRRSV infection failed to upregulate the m^6^A modification level of SQSTM1 (Fig. 8F). MeRIP-qPCR analysis confirmed that STM2457 significantly suppressed the m^6^A modification of *SQSTM1* mRNA (Fig. 8G). This suppression led to a significant decrease in both *SQSTM1* mRNA levels and protein expression (Fig. 8H and I). Furthermore, co-IP assays revealed a significant reduction in the interaction between endogenous IKKε and SQSTM1 in the presence of STM2457 (Fig. 8J).

To further confirm the role of SQSTM1, small interfering RNA (siRNAs) targeting *SQSTM1* were designed. Western blot analysis showed that SQSTM1 knockdown inhibited PRRSV N protein expression (Fig. S7A), while RT-qPCR confirmed reduced PRRSV *ORF7* transcription (Fig. S7B). The replication capacity of progeny viruses was also significantly reduced following SQSTM1 knockdown (Fig. S7C). Additionally, the interaction between IKKε and SQSTM1 was significantly diminished in siRNA-treated cells (Fig. 8K). Western blot analysis showed that the degradation of IKKε induced by PRRSV or METTL3 was prevented when SQSTM1 was silenced (Fig. 8L). Collectively, these findings suggest that SQSTM1-mediated IKKε degradation is dependent on the methylase activity of METTL3.

## DISCUSSION

During viral infection, the innate immune system recognizes self-derived (cellular) and non-self-derived (viral) nucleic acids, subsequently initiating antiviral innate immune responses to restrict viral replication (38). m^6^A is the most prevalent epigenetic modification in eukaryotic mRNA, playing dual roles in antiviral or proviral activities by regulating IFN-I responses (39). For instance, in vesicular stomatitis virus (VSV)-infected cells, increased m^6^A modification of viral-encoded transcripts reduced viral dsRNA formation, decreasing antiviral immune responses by lowering the efficacy of dsRNA recognition by RNA sensors such as RIG-I and MDA5 (40). Similarly, during human metapneumovirus infection, m^6^A modification on viral RNA mimics cellular RNA to evade RIG-I recognition, facilitating viral replication (41). Conversely, reduced m^6^A modification in the viral RNA of severe acute respiratory syndrome coronavirus 2 (SARS-CoV-2) enhanced RIG-I receptor binding and recognition, promoting downstream antiviral innate immune responses (42). Additionally, increased m^6^A modification on antiviral transcripts has been shown to enhance IFN-I responses (43, 44). In this study, PRRSV SD16 strain genomic transcripts were found to harbor m^6^A modifications, particularly enriched in nsp1α, nsp2a, nsp7b, nsp10, and ORF7 regions (Fig. 1C). Furthermore, our findings revealed that infection with the PRRSV SD16 strain significantly reprograms the host m^6^A epitranscriptomic landscape (Fig. 1D and E). Kyoto Encyclopedia of Genes and Genomes (KEGG) pathway enrichment analysis of genes with altered m^6^A methylation indicated substantial involvement in key signaling pathways, including PI3K-Akt and MAPK (Fig. 1F)—a pattern also observed in infections with the PRRSV HUN4 strain (45). These results suggest that both viral and host RNAs bearing m^6^A modifications may participate in the regulation of innate immune responses during PRRSV infection, an area that warrants further investigation.

METTL3, the core catalytic component of the m^6^A methyltransferase complex, catalyzes the majority of m^6^A modifications in eukaryotic RNA (42). PRRSV infection markedly increased METTL3 expression and altered its subcellular localization (Fig. 2A–D). These observations are consistent with findings from other positive-strand RNA viruses, such as enterovirus 71 and SARS-CoV-2 (46, 47), suggesting that modulation of METTL3 expression and distribution may be a common RNA viral strategy to enhance replication. Further analysis demonstrated that METTL3 serves as a critical regulator of the innate immune response during PRRSV infection by inhibiting IFNB1 production. This immunosuppressive effect appears to be mediated through disruption of the RIG-I–MAVS–TBK1/IKKε–IRF3 signaling cascade, in which METTL3 plays a regulatory role, particularly at the level of TBK1 and IKKε (Fig. 4A through G). The TBK1/IKKε complex is a key adapter in antiviral innate immune signaling, with both proteins phosphorylating IRF3 to promote IFN-I production and combat viral infections (48). Previous studies showed that METTL3 interacts with TBK1 during VSV infection. Furthermore, the amino acids 351-408 of METTL3 are crucial for its interaction with TBK1 (44). Our results indicate that METTL3 interacts not only with TBK1 but also with its associated kinase, IKKε (Fig. 4K through O). Notably, PRRSV-induced METTL3 selectively promotes the degradation of IKKε while leaving TBK1 protein levels unaffected (Fig. 5A through D). Structural domain analysis revealed that the kinase domain and ubiquitin-like domain of TBK1, along with the ubiquitin-like domain C and coiled-coil domains of IKKε, are critical for their interaction with METTL3 (Fig. 5G; Fig. S4B). This domain-specific binding may underlie the selective regulation of IKKε by METTL3. These findings suggest that PRRSV exploits a targeted immune evasion mechanism by modulating METTL3 activity to specifically degrade IKKε—disrupting host antiviral signaling without broadly compromising immune functions, thereby minimizing excessive host defense activation.

Viruses often exploit autophagy to their advantage (49). Previous studies have shown that PRRSV induces autophagy to benefit its replication (50). SQSTM1, an autophagy cargo receptor, recognizes and degrades substrates marked by ubiquitin (51–53). We found that both METTL3 overexpression and PRRSV infection enhanced the recognition of IKKε by the autophagy cargo receptor SQSTM1 (Fig. 7D and E). Importantly, the methyltransferase activity of METTL3 was critical for this process, as only the catalytically active form of METTL3 facilitated the SQSTM1–IKKε interaction (Fig. 7I). MeRIP-qPCR assays demonstrated that METTL3 directly regulates the m^6^A modification of *SQSTM1* mRNA (Fig. 8D through G), which in turn modulates its transcription and protein expression levels (Fig. 8H and I), as well as its ability to bind IKKε (Fig. 8J). These findings highlight a functional link between RNA epigenetic modification and selective autophagy. The involvement of m^6^A modification in autophagy regulation has been previously reported, indicating a broader biological role for this epitranscriptomic mark. For example, in renal lipid metabolism disorders and fibrosis, the antidiuretic drug cagliflozin, an SGLT2 inhibitor, reduces FTO to stabilize the m^6^A modification and the transcriptional level of *SQSTM1* mRNA, which induces the formation of autophagosomes and thus alleviates symptoms (54). In addition, reduction of the m^6^A-reading protein YTHDC1 in diabetic skin keratinocytes leads to attenuation of *SQSTM1* nuclear mRNA, which inhibits autophagy (55). In this study, we demonstrated that PRRSV infection modulates the autophagy pathway through METTL3-mediated m^6^A modification and, more importantly, uncovered its specific targeting of the RIG-I signaling cascade. This discovery expands the current understanding of how epitranscriptomic regulation intersects with innate immune evasion, positioning m^6^A modification as a pivotal node in viral strategy. Our findings reveal a novel mechanism by which PRRSV hijacks the SQSTM1-dependent autophagy pathway via METTL3-mediated m^6^A modification to selectively degrade IKKε, thereby linking m^6^A epigenetic control with autophagic degradation to dampen antiviral immunity.

Current PRRSV prevention strategies, including inactivated and live-attenuated vaccines, have proven insufficient in fully preventing outbreaks and controlling viral spread (3). Effective antiviral drug development provides new opportunities to combat PRRSV. Several small molecules have demonstrated promising anti-PRRSV effects in previous studies. For instance, baicalin, a natural flavonoid, inhibits PRRSV replication by directly interacting with viral particles (4). Sangenon C, a natural compound, inhibits the activation of the NF-κB signaling pathway by promoting TRAF2 expression, thereby reducing PRRSV replication (56). Nitazoxanide significantly inhibits PRRSV proliferation and transmission by enhancing IFNB1 production (57). This study elucidates a molecular mechanism by which PRRSV evades host innate immunity through the METTL3-mediated m^6^A-autophagy axis. Pharmacological inhibition of METTL3 with STM2457 (Fig. S5C and D) and blockade of autophagy with 3-MA (Fig. 6E) significantly reduced PRRSV replication *in vitro*. These findings suggest that STM2457 and 3-MA represent promising candidates for antiviral intervention against PRRSV. However, further *in vivo* studies in pigs are essential to evaluate their therapeutic potential and safety profiles.

**Fig 9:**
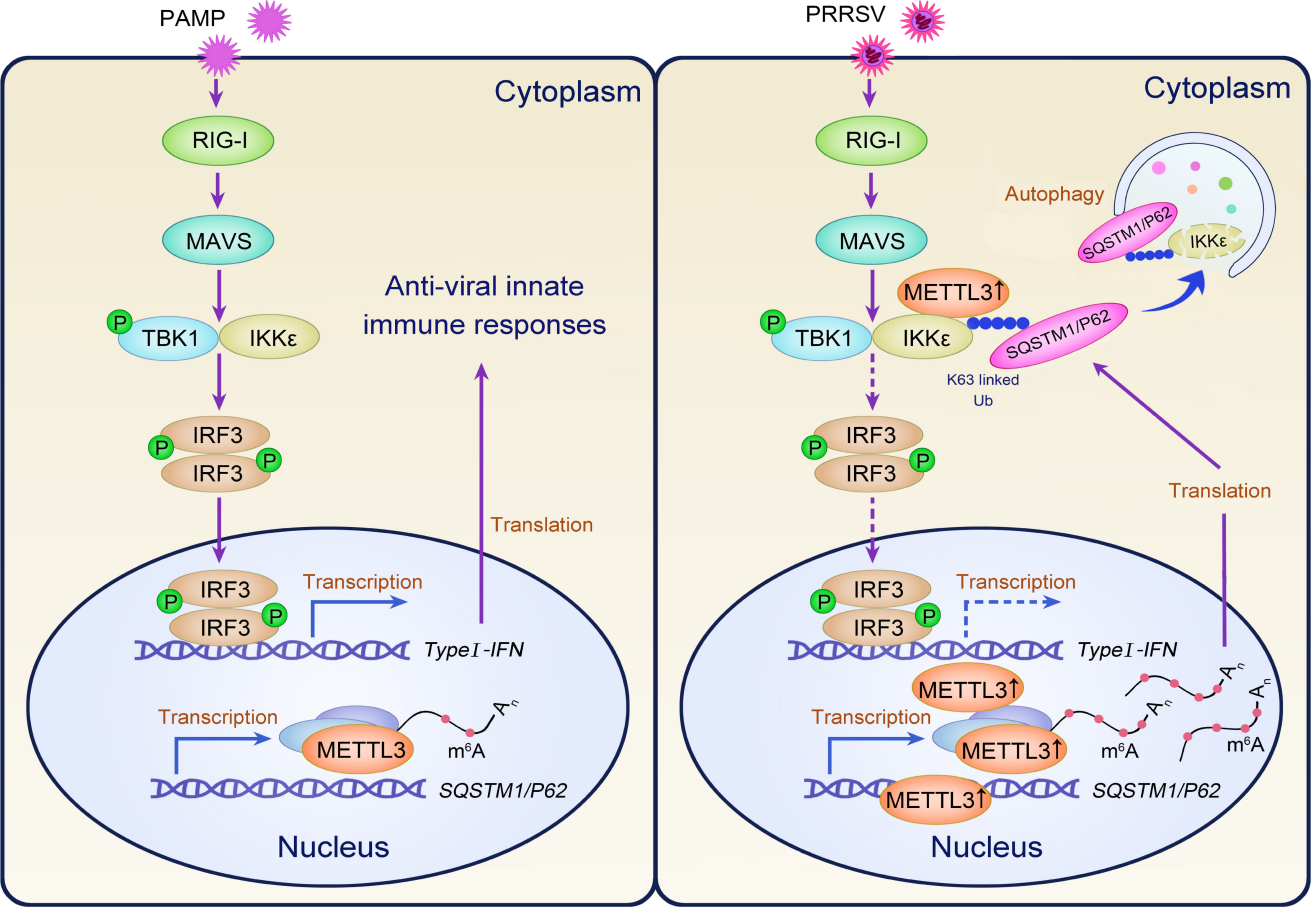
Schematic model of METTL3-mediated PRRSV immune evasion. Upon PRRSV infection, METTL3 protein expression is upregulated, resulting in its translocation from the nucleus to the cytoplasm. In the cytoplasm, METTL3 interacts with the TBK1/IKKε complex and promotes K63-linked ubiquitination of IKKε. This ubiquitination facilitates recognition by the autophagy cargo receptor SQSTM1, leading to IKKε degradation. Simultaneously, METTL3 enhances m^6^A modification of SQSTM1 mRNA, increasing its transcription and promoting autophagy. These mechanisms suppress *IFNB1* production, allowing PRRSV to evade host immunity and replicate efficiently.

In conclusion, our findings identify METTL3 as a critical modulator of innate immune responses during PRRSV infection. We demonstrated that METTL3 inhibits IFNB1 production by promoting IKKε degradation through SQSTM1-dependent autophagy, in both m^6^A-dependent and m^6^A-independent manners, thereby facilitating PRRSV replication (Fig. 9). This study highlights METTL3’s pivotal role in PRRSV immune evasion and positions it as a potential therapeutic target for anti-PRRSV drug development.

## MATERIALS AND METHODS

### Cells and viruses

MARC-145 and HEK293T cells were cultured in Dulbecco’s Modified Eagle Medium (DMEM, Gibco, Grand Island, NY, USA), supplemented with 10% heat-inactivated fetal bovine serum (FBS) and 100 U/mL penicillin, along with 100 µg/mL streptomycin sulfate.

The highly pathogenic PRRSV SD16 strain (GenBank: JX087437.1), provided by Professor Qin Zhao (Northwest A&F University, China), was used in all experiments unless otherwise specified. The virus was propagated and titrated in MARC-145 cells and stored at −80°C for subsequent analyses.

### Antibodies and reagents

The following antibodies were utilized in this study: anti-METTL3 (Abcam, Ab195352, USA), anti-Flag-HRP (Proteintech, HRP-66008, USA), anti-IKKi/IKKε (Abcam, Ab7891), anti-SQSTM1 (Proteintech, 66184-1-Ig), anti-LC3B (Abclonal, A19665, China), anti-IRF3 (Abways, CY5779), anti-pIRF3 (Abclonal, AP0995), anti-TBK1 (Abclonal, A3458), anti-pTBK1 (Abclonal, AP0847), anti-N6-methyladenosine (Abclonal, A19841), anti-Flag (Proteintech, 80010-1-RR), anti-HA (Proteintech, 81290-1-RR), anti-ATG5 (Abclonal, A0203), anti-ATG7 (Abclonal, A21895), and anti-β-actin (Proteintech, 20536-1-AP). Monoclonal anti-Flag M2 beads (M8823) were obtained from Sigma-Aldrich.

Chemical reagents were sourced as follows: 3-MA (3-methyladenine, HY-19312), MG132 (HY-13259), rapamycin (AY-22989), and STM2457 (HY-134836) from Med Chem Express, and Z-VAD-FMK (C1202) from Beyotime Institute of Biotechnology (Shanghai, China). Phenylmethylsulfonyl fluoride (PMSF) P8340 was procured from Solarbio Life Sciences (Beijing, China).

### Constructs

The swine *METTL3* (GenBank: XM_003128580.5), *TBK1* (GenBank: NM_001105292.1), *IKKε* (GenBank: XM_021063311.1), and *SQSTM1* (GenBank: XM_003123639.4) genes were amplified from PAM cells and cloned into p3xFlag-cmv-14, pCAGGS-HA, and pEGFP-N1 plasmids. The swine *RIG-I* (GenBank: NM_213804.2) gene was cloned into pCAGGS-HA plasmids, while swine *MAVS* (GenBank: NM_001097429.1) and *IRF3* (GenBank: NM_213770.1) were inserted into p3xFlag-cmv-14 plasmids. Truncated *METTL3* constructs were generated using the full-length *METTL3* sequence as a template and cloned into pCAGGS-HA plasmids. IFNB-luc and pRL-TK plasmids were purchased from Genomeditech (China). In accordance with the instructions provided by the manufacturer, PEI (Polyscience, Illinois, USA) was used to transfect WT-Ub-HA, K48O-Ub-HA, or K63O-Ub-HA plasmids from the International Joint Research Center of National Animal Immunology. All plasmid constructs were verified by DNA sequencing.

### Construction of stable cell lines and lentivirus infection

Lentiviruses were produced using PEI and either empty vector or target gene plasmids, along with two packaging plasmids (psPAX2 and pMD2.G). Lentiviral particles were generated in HEK293T cells, filtered after a 48 h incubation, and added to MARC-145 cells in the presence of polymyxin (8 µg/mL, Solarbio, H8641, China). Cells were selected with puromycin (8 µg/mL, Solarbio, IP1160, China) for 5 days. Stable cell lines were confirmed by western blot using specific antibodies. The sequences employed in the present study are presented in Table S1.

### RNA-mediated interference

siRNA targeting *SQSTM1* (*siSQSTM1*) and *IRF3* (*siIRF3*), along with negative control siRNA (NC), were designed by Shanghai GenePharma Co., Ltd. Transient gene silencing was performed by transfecting siRNA into MARC-145 cells using Lipofectamine 2000 (Invitrogen, USA). Sequences used in this study are listed in Table S2.

### Reverse transcription-quantitative PCR

Total RNA was extracted to quantify the mRNA expression of target genes using RT-qPCR. *β-actin* was used as the internal normalization control. Primer sequences for RT-qPCR are shown in Table S3.

### Dual-luciferase reporter assay

HEK293T cells were seeded into 48-well plates and cultured overnight until reaching 80% confluence. Cells were co-transfected with METTL3 or control vector plasmids, along with IFNB-luc (100 ng/well) and pRL-TK (25 ng/well) plasmids as internal controls. After 24 h, cells were lysed, and luciferase activity was measured using a dual-luciferase reporter assay kit (TransGen Biotech, China), following the manufacturer’s instructions. Relative luciferase activity was calculated as the ratio of firefly luciferase to Renilla luciferase activity.

### Immunoprecipitation and western blot analysis

After 24 h of transfection, whole-cell extracts were prepared for co-IP in M2 buffer containing PMSF, 0.5% (vol/vol) Nonidet P-40, 20 mM Tris-HCl, 3 mM EDTA, and 3 mM EGTA. Cell lysates were centrifuged at 12,000 × *g* for 10 min, and supernatants were incubated with 10 µL monoclonal anti-Flag M2 beads for 3 h. Beads were washed five times with elution buffer, which contained 0.5% (vol/vol) Nonidet P-40, 20 mM Tris-HCl, 3 mM EDTA, 3 mM EGTA, and 500 mM NaCl, and boiled in 1% (wt/vol) SDS sample buffer to elute bound proteins. Western blot analysis was conducted on SDS-extracted cell lysates. Protein samples were resolved by SDS-PAGE and transferred onto nitrocellulose membranes, which were probed with appropriate antibodies for detection.

### Immunofluorescence assay

For IFA analysis, MARC-145 cells were transfected with the plasmid and incubated at 37°C for 36 h. The cells were then fixed with 4% buffered paraformaldehyde for 10 min and permeabilized with 0.1% Triton X-100 at room temperature for 5 min. Afterward, the cells were incubated with antibodies specific to target proteins at room temperature, followed by staining with goat anti-rabbit IgG (H&L) (1:500) and goat anti-mouse IgG (H&L) (1:500) for 1 h in the dark. The cells were washed three times with PBS, and the nuclei were counterstained with DAPI (1:5,000). Immunofluorescence was observed using a fluorescence microscope, with uninfected cells serving as controls for background staining (28).

### RNA extraction and sequencing

Total RNA was extracted from PRRSV-infected MARC-145 cells for RNA sample preparation. mRNA was purified from total RNA using poly-T oligo-attached magnetic beads. Fragmentation of mRNA was performed to synthesize first-strand complementary DNA for library preparation. Qualified libraries were pooled and sequenced on an Illumina NovaSeq 6000 platform to generate paired-end 150 bp reads. The sequencer-generated image data were converted into sequence data (reads) using CASAVA for base calling. Raw data underwent filtration to remove adapter sequences, reads with “N” bases, and low-quality reads, resulting in high-quality clean reads. These clean reads were mapped to the *Chlorocebus sabaeus* genome (Vero_WHO_p1.0, GenBank: GCF_015252025.1) and the PRRSV SD16 strain genome using Hisat 2 (v2.0.5).

### Methylated RNA immunoprecipitation sequencing

A total of 300 µg of total RNA was extracted, and mRNA was purified using poly-T oligo-attached magnetic beads (58, 59). RNA was fragmented into ~100 bp fragments and incubated with anti-m^6^A polyclonal antibodies (Synaptic Systems, 202-003) in IP buffer at 4°C for 2 h. The immunoprecipitated mRNA or input RNA was used to construct libraries with the NEBNext Ultra RNA Library Prep Kit for Illumina (New England Biolabs). Libraries were sequenced on an Illumina NovaSeq or HiSeq platform with paired-end 150 bp reads.

m^6^A peak calling of host and viral epitranscriptomes was performed using the exomePeak R package (v2.16.0) for host genes and MACS2 (v2.0) for viral genes, with *P* < 0.05 and fold change >1 as thresholds. Peaks were mapped to 5′-UTR, CDS, and 3′-UTR regions to assess their distribution. Sequences used in this study are listed in Table S4.

### Data analysis

Gene expression analysis was performed by counting read numbers using featureCounts v1.5.0-p3. The expression level of each gene was estimated using the fragments per kilobase of transcript per million mapped reads method. Differential expression analysis between groups was conducted using the DESeq2 R package (1.20.0), which employs a negative binomial distribution model to determine differential gene expression. *P*-values were adjusted for the false discovery rate using the Benjamini-Hochberg method. Genes with adjusted *P*-values (Padj) <0.05 and log_2_ (fold change) >1 were considered significantly differentially expressed.

Gene Ontology (GO) enrichment analysis for differentially expressed genes was performed using the ClusterProfiler R package (Version 3.8.1), correcting for gene length bias. GO terms with corrected *P*-values <0.05 were considered significantly enriched. KEGG pathway enrichment analysis was performed to identify high-level functions of differentially expressed genes using the KEGG database (http://www.genome.jp/kegg/) and the ClusterProfiler R package 3.8.1. *P*-values <0.05 were considered significant.

### Viral titration

Viral infectivity was determined using the TCID_50_ assay. On day 0, cells were seeded at a density of 1 × 10⁴ cells per well in 96-well plates. On day 1, cells were infected with serially diluted viruses and incubated at 37°C for 2 h. After incubation, the viral inoculum was removed, and cells were washed with PBS. Maintenance media (100 µL; 2% FBS/DMEM) was added to each well, and cells were incubated for 5–7 days. The TCID_50_ value was calculated using the Reed-Muench method, with cytopathic effects monitored daily.

### Cytotoxicity assay

Cells were seeded into 96-well plates and grown in DMEM supplemented with 10% FBS. Once cells reached confluence, compounds were diluted to varying concentrations in medium containing 2% FBS. Control wells contained DMEM with 0.5% DMSO. Treated cells were incubated at 37°C in a humidified atmosphere with 5% CO₂ for 48–72 h. Cell viability was assessed by measuring absorbance at 450 nm using a microplate reader.

### Statistical analysis

Data are presented as means ± standard errors. Statistical analysis was performed using GraphPad Prism 8 (GraphPad Software, USA). Significant differences compared to controls were determined at thresholds of *P < 0.05, **P < 0.01, ***P < 0.001, and ****P < 0.0001.

## Data Availability

All data generated or analyzed during this study are included in the published article. Sequencing data sets have been deposited in the NCBI GEO repository under submission ID GSE281664.

## SUPPLEMENTAL MATERIAL

Supplemental figures jvi.00098-25-s0001.docx Fig. S1 to S7.

Table S1 jvi.00098-25-s0002.docx sgRNA sequences used in this study.

Table S2 jvi.00098-25-s0003.docx siRNA sequences used in this study.

Table S3 jvi.00098-25-s0004.docx RT-qPCR sequences used in this study.

Table S4 jvi.00098-25-s0005.docx Primers used for MeRIP-qPCR in this study.

ASM does not own the copyrights to Supplemental Material that may be linked to, or accessed through, an article. The authors have granted ASM a non-exclusive, world-wide license to publish the Supplemental Material files. Please contact the corresponding author directly for reuse.
