# Supplementary material for: METTL3 regulates PRRSV replication by suppressing interferon beta through autophagy-mediated IKKε degradation
Source: J Virol. 2025 Jun 23;99(7):e00098-25. doi: 10.1128/jvi.00098-25 (PMC12282061; doi:10.1128/jvi.00098-25)
Supplement: Supplemental figures — Fig. S1 to S7. [file jvi.00098-25-s0001.docx]

**Supporting information**

**
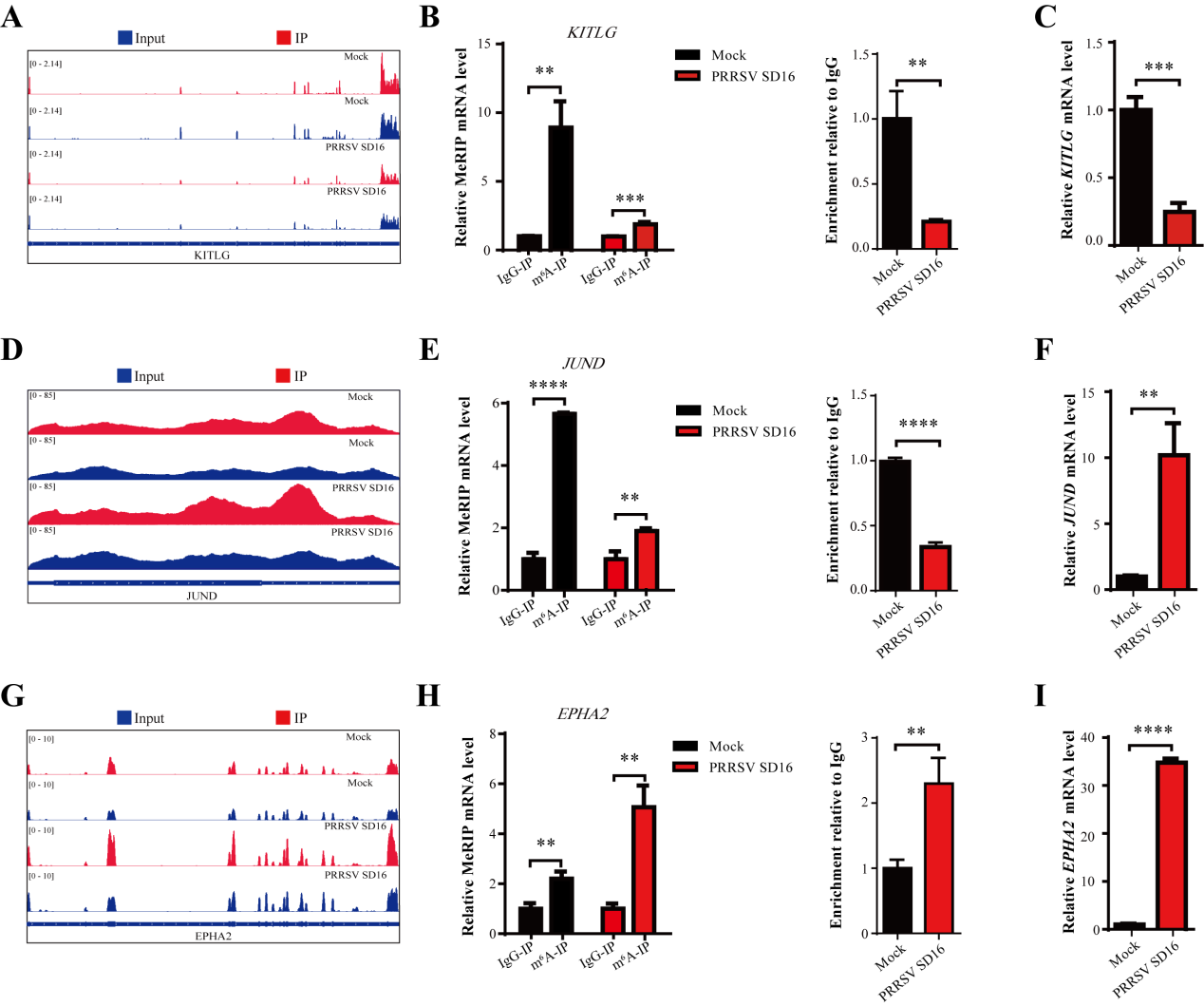
**

**Fig. S1. MeRIP-qPCR detection of m^6^A modification levels in KITLG, JUND, and EPHA2. (A)** IGV visualization of m^6^A peaks in *KITLG* transcripts from PRRSV-infected and uninfected MARC-145 cells. **(B and C)** After infecting MARC-145 cells with PRRSV (MOI = 0.5) for 36 h, total cellular RNA was extracted, and the m^6^A modification levels of *KITLG*, were detected by MeRIP-qPCR (B) and RT-qPCR (fold changes normalized to *β-actin*) (C). **(D-F)** JUND. **(G-I)** EPHA2.


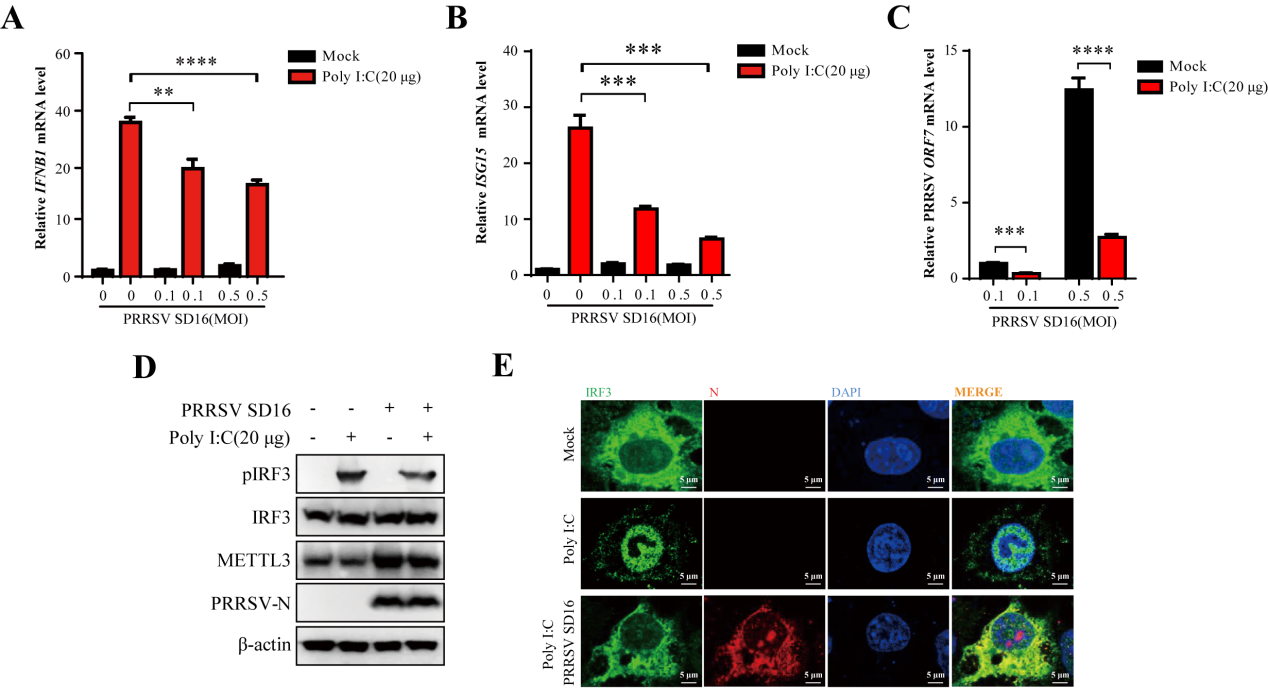


**Fig. S2** **PRRSV negatively regulates innate immunity. (A-C)** MARC-145 cells were infected by PRRSV with MOI = 0, 0.1 or 0.5, respectively. 24 h later, the cells were treated with 20 μg of Poly I:C. Cells were collected 12 h later to detect transcription of *IFNB1* (A), *ISG15* (B), and PRRSV *ORF7* using RT-qPCR (fold changes normalized to *β-actin*). **(D)** MARC-145 cells were infected with PRRSV (MOI = 0.5). 24 h later they were treated with 20 μg of Poly I:C. Cells were collected 12 h later for western blot to detect pIRF3, IRF3, METTL3 and PRRSV N protein changes. **(E)** Immunofluorescence microscopy of IRF3 subcellular localization in PRRSV-infected MARC-145 cells treated with Poly I:C. Cells were fixed 12 h later and analyzed for subcellular localization of IRF3 and PRRSV N proteins by IFA. Cell nuclei were counterstained with DAPI. Fluorescence images were acquired by confocal laser scanning microscopy.


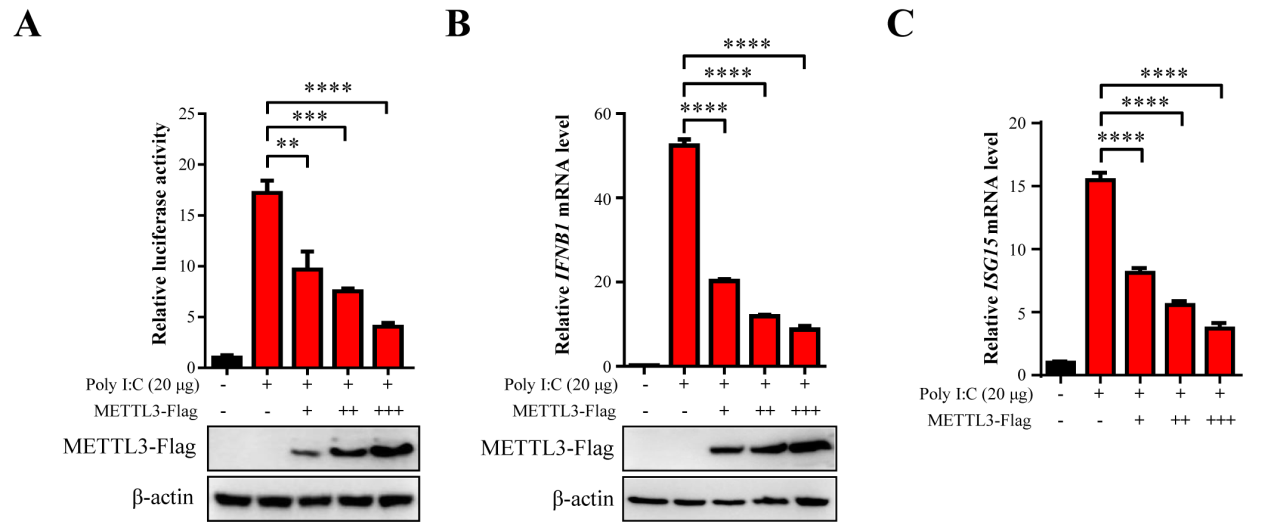


**Fig. S3 METTL3 negatively regulates *IFNB1* mRNA production.** **(A)** HEK293T cells were co-transfected with IFNB-Luc (100 ng), pRL-TK (25 ng), and varying amounts of METTL3-Flag plasmid (100, 200, 250 ng). An empty vector served as a negative control. After 12 h of transfection, cells were treated with 20 µg/mL Poly I:C for an additional 12 h. Dual-luciferase assays were performed to measure promoter activity. **(B and C)** MARC-145 cells transfected with METTL3-Flag (100, 200, or 250 ng) or an empty vector were treated with 20 µg/mL Poly I:C for 12 h. Western blot analysis was used to detect METTL3-Flag expression, and RT-qPCR was conducted to evaluate *IFNB1* (B) and *ISG15* (C) transcription (fold changes normalized to *β-actin*).


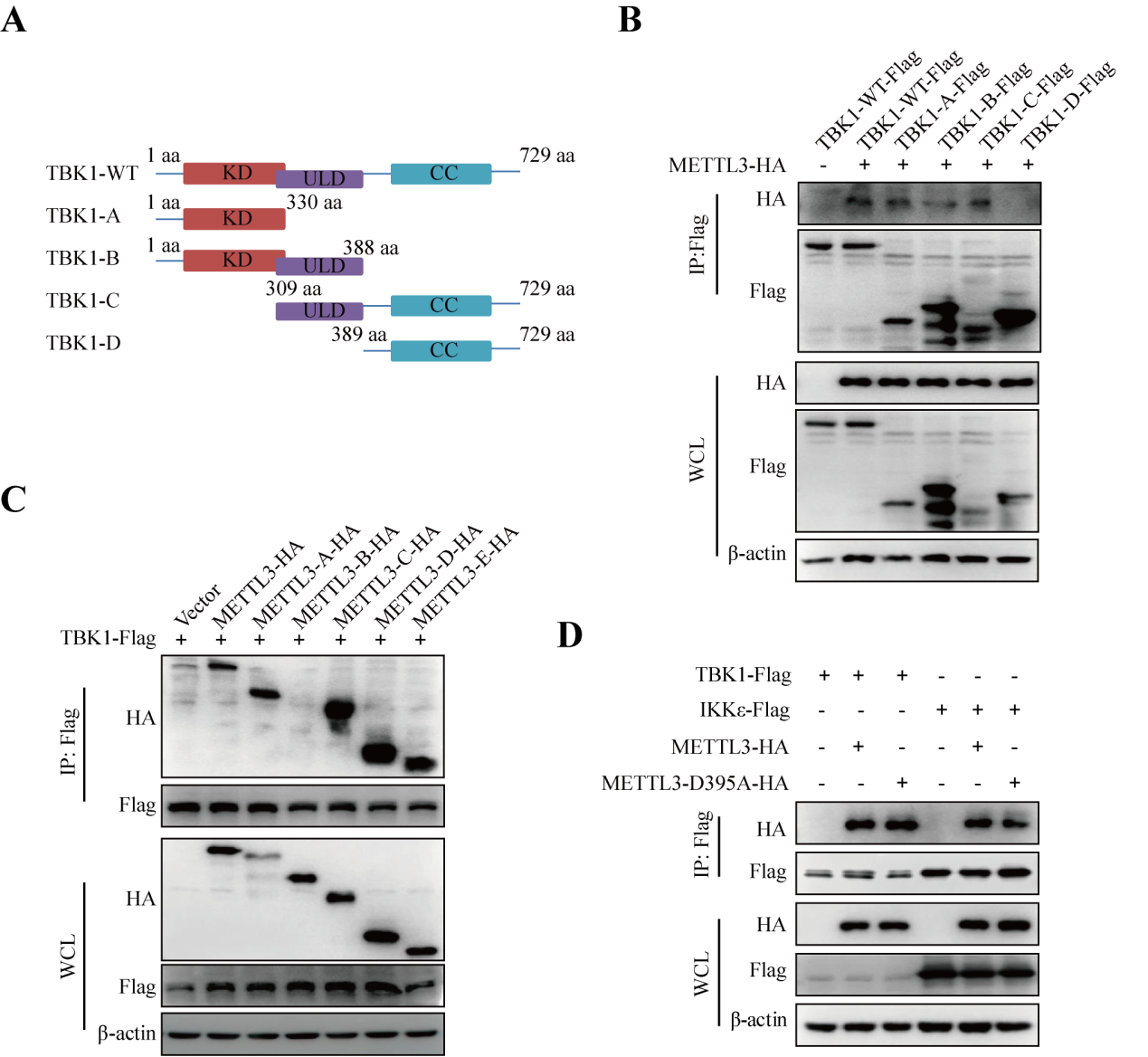


**Fig. S4** **Key structural domains of METTL3 interacting with TBK1.** **(A)** Schematic representation of Flag-tagged truncated TBK1 constructs. **(B)** HEK293T cells were co-transfected with METTL3-HA and its truncated mutant plasmids along with TBK1-Flag. Co-IP assays were performed at 24 h. **(C)** HEK293T cells were co-transfected with TBK1-Flag and its truncated mutant plasmids along with METTL3-HA. Co-IP assays were performed at 24 h. **(D)** Co-IP analysis of TBK1-Flag, IKKε-Flag, METTL3-HA, and METTL3-D395A-HA interaction in HEK293T cells transfected with the indicated plasmids. After 24 h, lysates were immunoprecipitated with an anti-Flag antibody and analyzed by western blot.


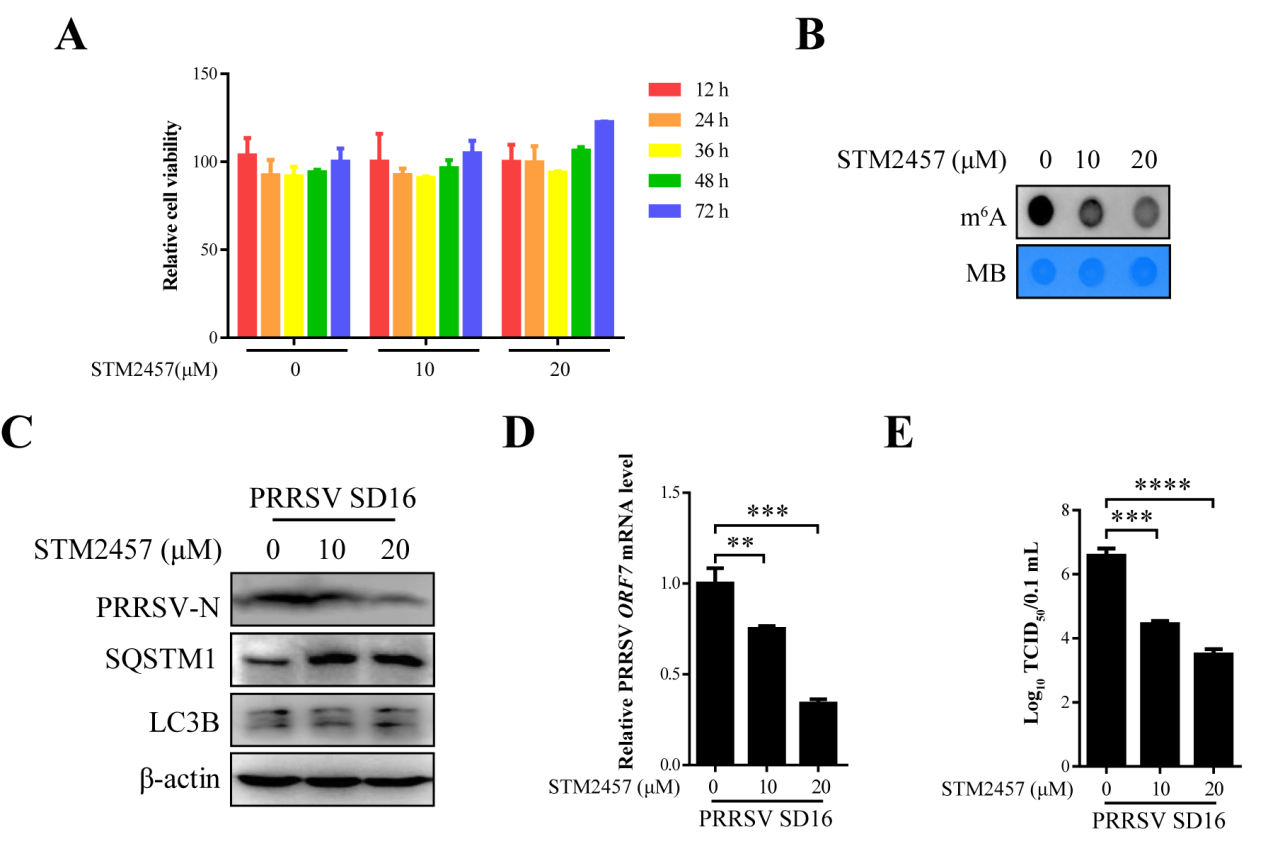


**Fig. S5** **STM2457 inhibits PRRSV replication. (A)** MARC-145 cells were treated with STM2457 (0, 10, 20 μM) for 12-72 h. Cell viability was assessed using the CCK-8 cytotoxicity assay. **(B)** Dot blot was used to detect relative m^6^A levels in MARC-145 cells treated with STM2457 (0, 10, 20 μM), m^6^A means anti-m^6^A dot blot, MB means methylene blue staining. **(C-E)** MARC-145 cells were treated with STM2457 (0, 10, 20 μM) for 4 h, then infected with PRRSV (MOI = 0.5), and the cells were collected after 36 h. The cells were subjected to western blot (C), RT-qPCR (D), and TCID_50_ assays (E).


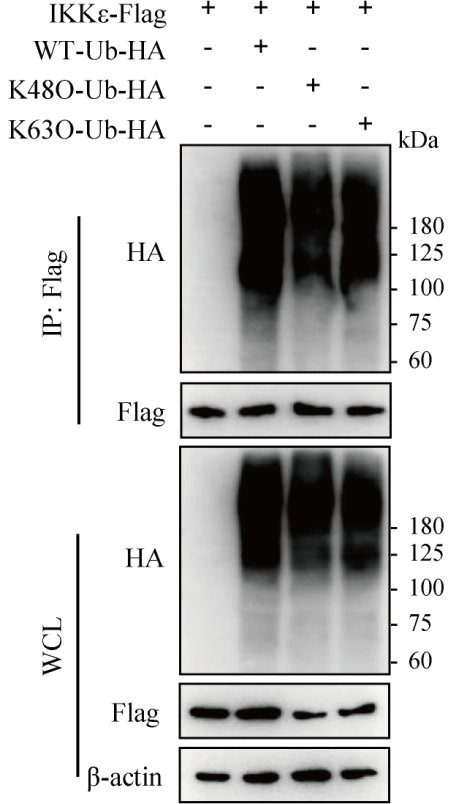


**Fig. S6 HEK293T cells were co-transfected with WT-Ub-HA, K48O-Ub-HA, and K63O-Ub-HA plasmids along with IKKε-Flag plasmid. 24 h later, Co-IP analysis was performed.**

**
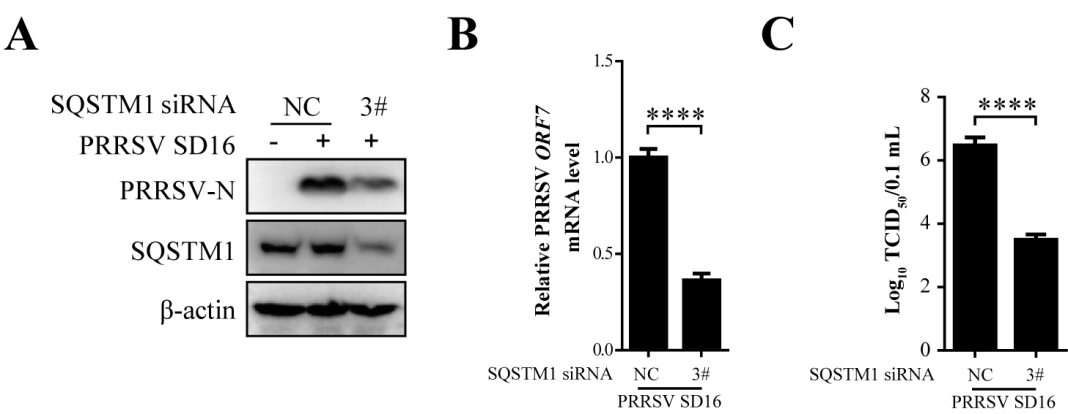
**

**Fig. S7 SQSTM1 knockdown inhibits PRRSV replication.** **(A-C)** MARC-145 was transfected with SQSTM1 siRNA3 or NC for 24 h, infected with PRRSV (MOI = 0.5), and cells were collected at 36 h for western blot (A), RT-qPCR (B), and TCID_50_ assay (C).
