# Supplementary material for: METTL3 regulates PRRSV replication by suppressing interferon beta through autophagy-mediated IKKε degradation
Source: J Virol. 2025 Jun 23;99(7):e00098-25. doi: 10.1128/jvi.00098-25 (PMC12282061; doi:10.1128/jvi.00098-25)
Supplement: Table S1 — sgRNA sequences used in this study. [file jvi.00098-25-s0002.docx]

**Table S1.** The sequences of sgRNA used in this study.

| Name | | Forward sequence (5ʹ-3ʹ) | | Reverse sequence (5ʹ-3ʹ) | |
| --- | --- | --- | --- | --- | --- |
| *sgMETTL3-1* | CACCGAGGCGGAAGCAGGACTCGG | | AAACCCGAGTCCTGCTTCCGCCTC | |  |
| *sgMETTL3-2* | CACCGGCGTCTACAAGCAACATGG | | AAACCCATGTTGCTTGTAGACGCC | |  |
| *sgMETTL3-3* | CACCGCTACTGCACCCACCTCTGG | | AAACCCAGAGGTGGGTGCAGTAGC | |  |
| *sgIKKε-1*  *sgIKKε-2*  *sgIKKε-3*  *sgAtg5-1*  *sgAtg5-2*  *sgAtg5-3*  *sgAtg7-1*  *sgAtg7-2*  *sgAtg7-3* | CACCGTGTGGGTCCTAAGGGCCTT  CACCGCAGCAAGCCCTGAAAGTTC  CACCGCTCAGGGCAGCTCAGAGTG  CACCGAAGAGTAAGTTATTTGACGT  CACCGCCTTAGATGGACAGTGCAGA  CACCGATCACAAGCAACTCTGGAT  CACCGCTTGAAAGACTCGAGTGTGT  CACCGTCCTACTTTAGACTTGGACA  CACCGCCAGAAAATATTCCCCGGTG | | AAACAAGGCCCTTAGGACCCACAC  AAACGAACTTTCAGGGCTTGCTGC  AAACCACTCTGAGCTGCCCTGAGC  AAACACGTCAAATAACTTACTCTTC  AAACTCTGCACTGTCCATCTAAGGC  AAACATCCAGAGTTGCTTGTGATC  AAACACACACTCGAGTCTTTCAAGC  AAACTGTCCAAGTCTAAAGTAGGAC  AAACCACCGGGGAATATTTTCTGGC | |  |
