# Supplementary material for: METTL3 regulates PRRSV replication by suppressing interferon beta through autophagy-mediated IKKε degradation
Source: J Virol. 2025 Jun 23;99(7):e00098-25. doi: 10.1128/jvi.00098-25 (PMC12282061; doi:10.1128/jvi.00098-25)
Supplement: Table S2 — siRNA sequences used in this study. [file jvi.00098-25-s0003.docx]

**Table S2.** The sequences of siRNAs used in this study.

| Name | | Forward sequence (5ʹ-3ʹ) | | Reverse sequence (5ʹ-3ʹ) |
| --- | --- | --- | --- | --- |
| si*IRF3-3* | UAGGGCACUGCCACACAUATT | | UAUGUGUGGCAGUGCCCUATT | |
| si*SQSTM1-3* | GCUCCUGCAGACCAAGAAUTT | | AUUCUUGGUCUGCAGGAGCTT | |
