# Supplementary material for: METTL3 regulates PRRSV replication by suppressing interferon beta through autophagy-mediated IKKε degradation
Source: J Virol. 2025 Jun 23;99(7):e00098-25. doi: 10.1128/jvi.00098-25 (PMC12282061; doi:10.1128/jvi.00098-25)
Supplement: Table S3 — RT-qPCR sequences used in this study. [file jvi.00098-25-s0004.docx]

**Table S3.** The sequences of RT-qPCR used in this study.

| Name | Forward sequence (5ʹ-3ʹ) | | Reverse sequence (5ʹ-3ʹ) |
| --- | --- | --- | --- |
| PRRSV *ORF7* | AGATCATCGCCCAACAAAAC | ACACAATTGCCGCTCACTA | |
| *ISG15* | GGTGCAAAGCTTCAGAGACC | GTCAGCCAGACCTCATAGGC | |
| *IFNβ* | GCTCTCCTGTTGTGCTTCTCCA | CAATAGTCTCATTCCAGCCAGTC | |
| *IKKε*  *β-actin* | GGCGGATTACAACACGGCTA  CGTGGACATCCGTAAAGAC | TGCAGCCCCCGTAACATTAG  GGAAGGTGGACAGCGAGGC | |
