# Supplementary material for: METTL3 regulates PRRSV replication by suppressing interferon beta through autophagy-mediated IKKε degradation
Source: J Virol. 2025 Jun 23;99(7):e00098-25. doi: 10.1128/jvi.00098-25 (PMC12282061; doi:10.1128/jvi.00098-25)
Supplement: Table S4 — Primers used for MeRIP-qPCR in this study. [file jvi.00098-25-s0005.docx]

**Table S4.** The primers used for MeRIP-qPCR in this study.

| Name | Forward sequence (5ʹ-3ʹ) | Reverse sequence (5ʹ-3ʹ) |
| --- | --- | --- |
| PRRSV-*Nsp1a* | CGGGTCGCAGCTGAAATCTA | ACTCACATGCAGGGAGTTGG |
| PRRSV-*Nsp2a* | TGGGTCAAAAGCTACCCGC | CAGGCTTGTCCTCTGGCAA |
| PRRSV-*Nsp7b* | TGGGACAAGAATTCCGGTGA | CTAAGGCACTCCCACTCGTC |
| PRRSV-*Nsp10* | TGTCCATGGTCAACACGACC | GGGTGAGGACTTGCCCATAC |
| PRRSV-*ORF7* | AAACCAGTCCAGAGGCAAGG | CAGACACAATTGCCGCTCAC |
| *KITLG* | AGCATTTAGACATTTCTGGAGCCAT | ATGGCTCCAGAAATGTCTAAA |
| *JUND* | CATCGACATGGACACGCAGG | GCTCCGTGTTCTGGCTCTTA |
| *EPHA2* | GGGCCATGTGAGGCTAGTG | GTTGCGGGCCTCTACAGTAA |
| *SQSTM1*-1 | ATGGTGCACCCCAATGTGAT | TCGTAGTCTGGACAGACGCT |
|  |  |  |
